# Safety and Immunogenicity of an Adjuvanted *Clostridioides difficile* Vaccine Candidate in Healthy Adults: A Randomized Placebo-Controlled Phase 1 Study

**DOI:** 10.1093/infdis/jiae466

**Published:** 2024-10-24

**Authors:** Isabel Leroux-Roels, Azhar Alhatemi, Magalie Caubet, Fien De Boever, Bertrand de Wergifosse, Mohamed El Idrissi, Guilherme S Ferreira, Bart Jacobs, Axel Lambert, Sandra Morel, Charlotte Servais, Juan Pablo Yarzabal

**Affiliations:** Center for Vaccinology, Ghent University, Ghent University Hospital, Ghent, Belgium; Center for Vaccinology, Ghent University, Ghent University Hospital, Ghent, Belgium; GSK, Rixensart, Belgium; Center for Vaccinology, Ghent University, Ghent University Hospital, Ghent, Belgium; GSK, Rixensart, Belgium; GSK, Rixensart, Belgium; GSK, Amsterdam, The Netherlands; Center for Vaccinology, Ghent University, Ghent University Hospital, Ghent, Belgium; GSK, Wavre, Belgium; GSK, Rixensart, Belgium; GSK, Rixensart, Belgium; GSK, Wavre, Belgium

**Keywords:** adjuvant, *Clostridioides difficile*, immunogenicity, safety, vaccine candidate

## Abstract

**Background:**

This study investigated the safety, reactogenicity, and immunogenicity in healthy subjects of a *Clostridioides difficile* vaccine candidate with/without adjuvant, targeting toxins A and B.

**Methods:**

In this first-in-human, phase 1, observer-blind study, subjects aged 18–45 years were randomized to receive F2 antigen (n = 10) or placebo (n = 10), and subjects aged 50–70 years to receive F2 antigen plus AS01 adjuvant (n = 45), F2 antigen (n = 45), or placebo (n = 30) in 2 doses 1 month apart. A subcohort (n = 40) received a third dose 15 months later. Solicited adverse events (AEs) were recorded for 7 days and unsolicited AEs for 30 days after each dose. Immunogenicity was assessed at baseline and after each dose.

**Results:**

Solicited AEs were transient and most frequent in subjects receiving F2 antigen plus AS01. No serious AEs were considered related to study vaccine. Immunogenicity was substantially higher in subjects receiving F2 antigen plus AS01 than subjects receiving F2 antigen alone. A third dose increased the immune response in subjects with baseline neutralization titers below the assay lower limit of quantitation.

**Conclusions:**

The GSK *C. difficile* vaccine candidate was immunogenic, especially when given with AS01, and was well tolerated with an acceptable safety profile.

**Clinical Trial Registration:**

NCT04026009.

## Teaser

This first-in-human, randomized, placebo-controlled study in healthy adult volunteers investigated a *Clostridioides difficile* vaccine candidate targeting toxins A and B. The vaccine candidate was immunogenic, especially when given with AS01 adjuvant, and was well tolerated with an acceptable safety profile.

*Clostridioides difficile* (previously *Clostridium difficile*) is an anaerobic gram-positive bacterium. Disruption of the normal gut microflora by antibiotic treatment facilitates germination of *C. difficile* spores in the human gut. *C. difficile* infection (CDI) is characterized by damage to the gut mucosa, which depends on 2 main toxins, toxin A (TcdA) and toxin B (TcdB) [1]. In humans, a strong immune response against TcdA has been associated with reduced disease severity [2] and decreased risk of recurrence [3]. Administration of the anti-TcdB neutralizing monoclonal antibody bezlotoxumab in addition to antibiotic treatment significantly reduced recurrence of CDI in a clinical trial; addition of the anti-TcdA antibody actoxumab did not improve efficacy [4].

CDI is a major cause of health care-associated diarrhea [5] and recent hospitalization is an important risk factor for recurrent CDI [6]. Older age, antibiotic use, proton pump inhibitor use, and comorbidities are risk factors for community-acquired and recurrent CDI [6, 7]. The impact of *C. difficile* ranges from asymptomatic carriage through mild diarrhea to life-threatening complications such as pseudomembranous colitis and toxic megacolon [2].

In the United States, the number of CDI in 2017 was estimated at 462 100 [8], with an estimated 4300 in-hospital deaths associated with community-associated CDI and 16 200 with healthcare-associated CDI [8]. CDI in US acute health care facilities was associated with excess costs estimated at US$4.8 billion in 2008 [9], not including indirect costs in acute-care facilities or the costs of managing CDI elsewhere in the health care system [9].

Antibiotics are currently the main treatment for CDI, recommended in treatment guidelines in the United States [10] and Europe [11]. Approximately 5%–40% of patients treated for CDI experience recurrent diarrhea, with risk of recurrence associated with age >65 years, increased severity of underlying disease, and exposure to additional antibiotics [5]. The anti-TcdB monoclonal antibody, bezlotoxumab, is recommended as adjunctive treatment with antibiotics for patients with recurrent CDI [10, 11], except for those with history of congestive heart failure and with caution in those with severe underlying cardiovascular comorbidities [12].

As *C. difficile* toxins are immunogenic, active immunization may be effective in preventing disease [2]. GSK developed a *C. difficile* vaccine candidate (GSK2904545A) containing the F2 antigen, a fusion protein comprising the C-terminal portions of TcdA and TcdB containing the toxin binding domains [2]. Adjuvants in vaccines aim to enhance the intrinsic immunogenicity of antigens by stimulating components of the innate immune system [13, 14]. The GSK *C. difficile* vaccine candidate investigated in this study contains AS01 adjuvant. AS01 has been shown to increase the immunogenicity of varicella zoster virus glycoprotein [15] and respiratory syncytial virus F protein [16].

The objective of this first-in-human study was to investigate the safety, reactogenicity, and immunogenicity of the GSK *C. difficile* vaccine candidate in healthy human subjects.

## METHODS

### Study Design and Population

**Figure 1.**
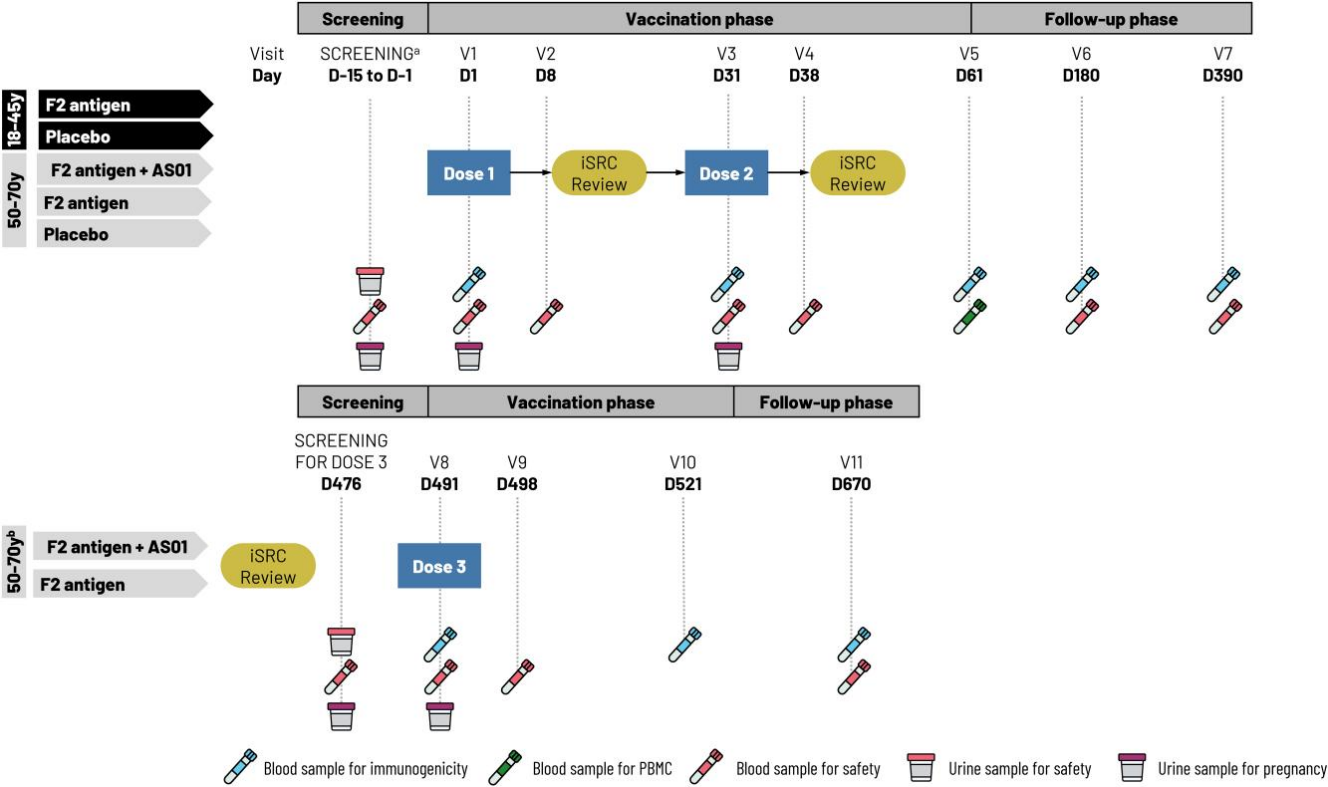
Study design. ^a^Visit 1 (day 1) was to be scheduled as soon as the results from hematology and biochemistry analysis were available. ^b^A subcohort of subjects who successfully completed all visits up to visit 7, received 2 doses on an active arm (ie, the subjects who received placebo were not to be considered for dose 3), did not meet any contraindication for subsequent vaccination, and still met the eligibility criteria received an additional dose, that is, dose 3. Abbreviations: AS01, liposome-based vaccine adjuvant system containing 2 immunostimulants: 3-*O*-desacyl-4′-monophosphoryl lipid A and the saponin QS-21; D, day; iSRC, internal safety review committee; PBMC, peripheral blood mononuclear cell; V, visit.

The study (NCT04026009) was conducted at a single center in Belgium between 5 August 2019 and 27 September 2022. This was a phase 1, first-in-human, single-center, randomized, observer-blind placebo-controlled study in healthy subjects with 2 or 3 treatment groups per step in a 4-step staggered design (Figure 1). All subjects received 2 doses of study treatment 1 month apart, at days 1 and 31. Visits occurred 7 days after each vaccine dose (days 8 and 38) and at days 61, 180, and 390 (Figure 1).

A subcohort of subjects aged 50–70 years who had received 2 doses of an active formulation were invited to receive a third dose of the same formulation at day 491, approximately 15 months after dose 2. The third dose was offered to all subjects who had received 2 vaccine doses (therefore, placebo recipients were excluded from this subcohort), and who had completed all visits up to visit 7, had no contraindications for subsequent vaccination, and who still met all the eligibility criteria. The decision to offer a third dose was taken after review of all safety data accumulated after the second dose, resulting in the 15-month time interval between second and third dose. Further visits for this cohort occurred at days 498, 521, and 670 (Figure 1).

The study was observer-blind because of the different appearance of the vaccine candidate and placebo. Subjects and the personnel evaluating safety and reactogenicity were unaware of the study treatment administered, except for the subcohort receiving a third dose, in whom the study was partial-blind as it was known that they would not receive placebo. The study duration was approximately 1 year for subjects receiving 2 doses, and approximately 2 years for subjects receiving 3 doses.

### Ethics

The study was conducted in accordance with the Declaration of Helsinki, Good Clinical Practice guidelines, and applicable laws and regulations. The protocol and all other relevant documents were approved by the independent ethics committee of the Ghent University Hospital in Belgium (reference 2019/0829 BC-05635, approval date 13 May 2020). Written informed consent was obtained from all subjects before study entry.

### Study Vaccine

The *C. difficile* vaccine candidate contained lyophilized F2 antigen, a fusion protein comprising the C-terminal portions of TcdA and TcdB. The F2 antigen contains the portion of TcdA from residue 2121 to residue 2686 and the portion of TcdB from residue 1968 to residue 2366 (numbering based on VPI10463 strain), with a junction between the second short repeating unit (SR) of SR cluster VIII of TcdA and the third SR of SR cluster II of TcdB (Supplementary Figure 1*A*). AS01 is a liposome-based vaccine adjuvant system containing 2 immunostimulants: 3-*O*-desacyl-4′-monophosphoryl lipid A and the saponin QS-21 (licensed by GSK from Antigenics, Inc, a wholly owned subsidiary of Agenus, Inc, a Delaware, US corporation). Placebo was 150 mM saline. Vaccine and placebo were administered by intramuscular injection. All subjects were monitored for 60 minutes after administration.

### Study Population

Healthy male or female participants were eligible if aged 18–45 years at dose 1 (step 1) or aged 50–70 years at dose 1 (steps 2, 3, and 4), free of uncontrolled chronic illness, and had provided informed consent (for further details, see Supplementary Methods).

### Safety and Reactogenicity Assessment

Solicited local and general adverse events (AEs) within 7 days of each dose of vaccine were recorded. Solicited local AEs were pain, swelling, or redness at the injection site. Solicited general AEs were fatigue, fever, gastrointestinal symptoms (nausea, diarrhea, vomiting, abdominal pain), headache, myalgia, shivering, or arthralgia.

Unsolicited AEs were recorded within 30 days of each dose and serious adverse events (SAEs) were recorded up to the end of the study, and classified according to Medical Dictionary for Regulatory Activities (MedDRA) version 22.0. Potential immune-mediated disorders (pIMD) (Supplementary Table 1) were recorded up to the end of the study. Occurrence of hematological (white blood cells, platelet count, and hemoglobin level) and biochemical (alanine aminotransferase, aspartate aminotransferase, creatinine, and uric acid) laboratory abnormalities were recorded as part of safety assessment.

The relationship of all AEs other than solicited local AEs to study treatment was assessed by the investigator using clinical judgment as “No” (no reasonable possibility that the AE was causally related to the study vaccine) or “Yes” (reasonable possibility that the AE was causally related to the study vaccine). All solicited local AEs were considered causally related to vaccination. Solicited local and general AEs, and hematology and biochemical abnormalities were graded based on published guidance [17]. Unsolicited AE and SAE intensity was categorized by the investigator as mild (easily tolerated, not interfering with normal everyday activities), moderate (interfering with normal everyday activities), or severe (preventing normal everyday activities).

### Immunogenicity Assessment

Serum neutralization activity against TcdA and TcdB was measured using optimized toxin neutralization assays (TNAs) on 2 human colonic cell lines, chosen for their relevant tissue origin and sensitivity to the toxins: HT-29 cell line for TcdA TNA, and HCT-116 cell line for TcdB TNA. (For details of the TNA, see Supplementary Methods). There is no international standard and no correlate of protection. For quality control, all assay runs included negative and positive controls to monitor assay performance, which consisted of human serum of known neutralization titer. All immunogenicity analyses were conducted at GSK's clinical laboratories at Rixensart, Belgium. The lower limits of quantitation (LLOQs) for TNAs measuring serum neutralization activity against TcdA and TcdB were 1:12 dilution (12 dil^−1^) and 1:15 dilution (15 dil^−1^), respectively. Enzyme-linked immunosorbent assays (ELISAs) were planned but not conducted as cross-reactivity (partial inhibition of anti-TcdB antibodies by TcdA antigen) was discovered, resulting in technical difficulties in developing specific assays.

### Statistical Analysis

All comparative analyses were exploratory. No formal statistical hypotheses were tested. All derivations, statistical analyses, summaries, graphs, and listings were generated using SAS version 9.4 or higher (SAS Institute, Inc). Missing data were not imputed. Safety and reactogenicity analyses were conducted on the exposed set (all subjects who received ≥1 dose of study treatment), and on the subcohort exposed set (all subjects who received 3 active doses). The percentage of participants reporting solicited and unsolicited AEs were tabulated with exact 95% confidence interval (CI).

Immunogenicity analyses were conducted on the per-protocol set (all subjects who received ≥1 dose of study treatment and had postvaccination immunogenicity data with no protocol deviations requiring exclusion). At each time point, the geometric mean titer (GMT) for neutralization activity was calculated with 95% CI.

## RESULTS

### Demographics

**Figure 2.**
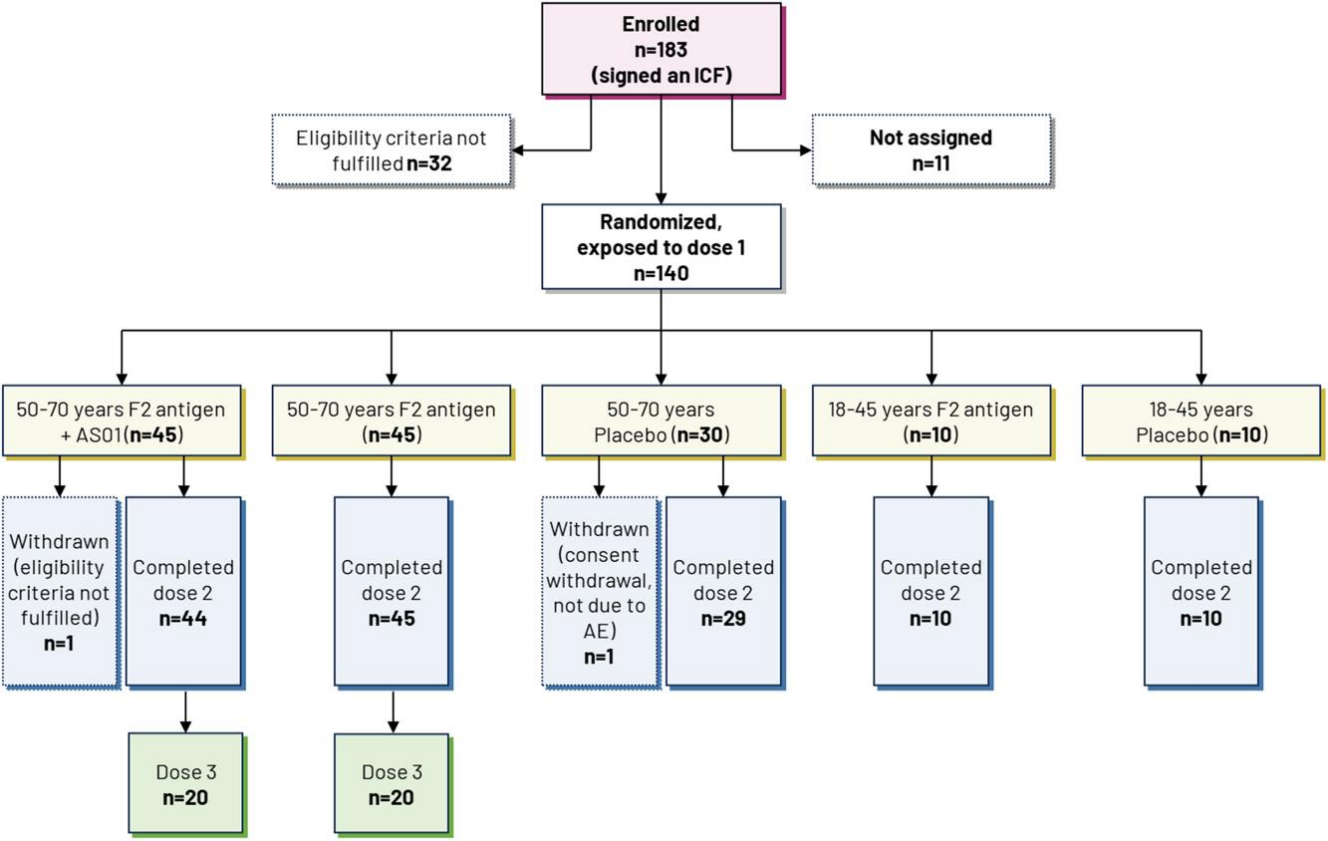
Subject disposition. Abbreviations: AE, adverse event; AS01, liposome-based vaccine adjuvant system containing 2 immunostimulants: 3-*O*-desacyl-4′-monophosphoryl lipid A and the saponin QS-21; ICF, informed consent form; n, number of subjects meeting criteria of status.

A total of 183 subjects were enrolled, of whom 140 were randomized and received ≥1 dose (exposed set). Of these, 138 completed the study and 2 withdrew prematurely (both aged 50–70 years, 1 receiving F2 antigen plus AS01 and 1 receiving placebo) (Figure 2). A total of 136 subjects received 2 doses of vaccine or placebo (1 subject did not receive the second dose due to an AE [fatigue] and 1 could not be contacted for the second dose), and 40 subjects received a third dose of vaccine (subcohort exposed set). Supplementary Table 2 presents the demographic characteristics of the exposed set.

### Safety and Reactogenicity

Supplementary Figure 2 and Supplementary Table 3 summarize solicited local and general AEs reported during 7 days after dose 1 and 2. The group receiving F2 antigen plus AS01 showed the most reactogenic profile. Most solicited AEs were mild/moderate with few reports of grade 3, and transient (median duration mostly ≤3 days) (Supplementary Table 4). The subcohort who received a third dose showed a similar pattern (Supplementary Figure 3, Supplementary Table 5, and Supplementary Table 6).

Among subjects aged 50–70 years, ≥1 unsolicited AE was reported by 27 (60.0%) subjects receiving F2 antigen plus AS01 (grade 3 in 3 subjects), 22 (48.9%) receiving F2 antigen (grade 3 in 2 subjects), and 11 (36.7%) receiving placebo (grade 3 in 1 subject). Among subjects aged 18–45 years, ≥1 unsolicited AE was reported by 3 (30.0%) receiving F2 antigen (grade 3 in 1 subject) and 2 (20.0%) receiving placebo (none grade 3). Eight subjects receiving F2 antigen plus AS01 reported ≥1 unsolicited AE assessed as causally related to vaccination, with none in the other groups. Among these eight, 3 had dizziness, 2 had lymphadenopathy, 2 had injection site pruritus, and 1 each had injection site warmth, injection site swelling, malaise, oral herpes, decreased appetite and paresthesia (subjects could have >1 condition). No grade 3 unsolicited AEs were assessed as causally related to vaccination.

In the subcohort who received 3 doses, ≥1 unsolicited AE was reported by 13 (65.0%) subjects receiving F2 antigen plus AS01 (grade 3 in 1 subject) and 9 (45.0%) receiving F2 antigen (grade 3 in 2 subjects). Four of the subjects receiving F2 antigen plus AS01 reported ≥1 unsolicited AE assessed as causally related to vaccination. Among these four, 2 each had lymphadenopathy, injection site pruritus and dizziness, and 1 each had injection site warmth, injection site swelling, and decreased appetite (subjects could have >1 condition). Lymphadenopathy is not unusual in vaccine trials, and both lymphadenopathy AEs in this study were mild/moderate and were not accompanied by any other symptoms that warranted further clinical investigation. No grade 3 unsolicited AEs were assessed as causally related to vaccination.

Seven subjects reported SAEs, 3 in the 50–70-year-old group receiving F2 antigen alone, 3 in the 50–70-year-old group receiving F2 antigen plus AS01 and 1 in the 18–45-year-old group receiving placebo. All the SAEs were considered not related to study vaccine. No fatal SAEs and no discontinuations due to AEs were reported. No SAEs or pIMDs were reported in the subcohort exposed set who received 3 doses. Two subjects in the 50–70-year-old group receiving F2 antigen reported pIMDs (Graves' disease and alopecia areata). Both were considered not related to study vaccine.

There were no findings related to the clinical laboratory parameters or vital signs considered to be clinically significant.

### Immunogenicity

**Figure 3.**
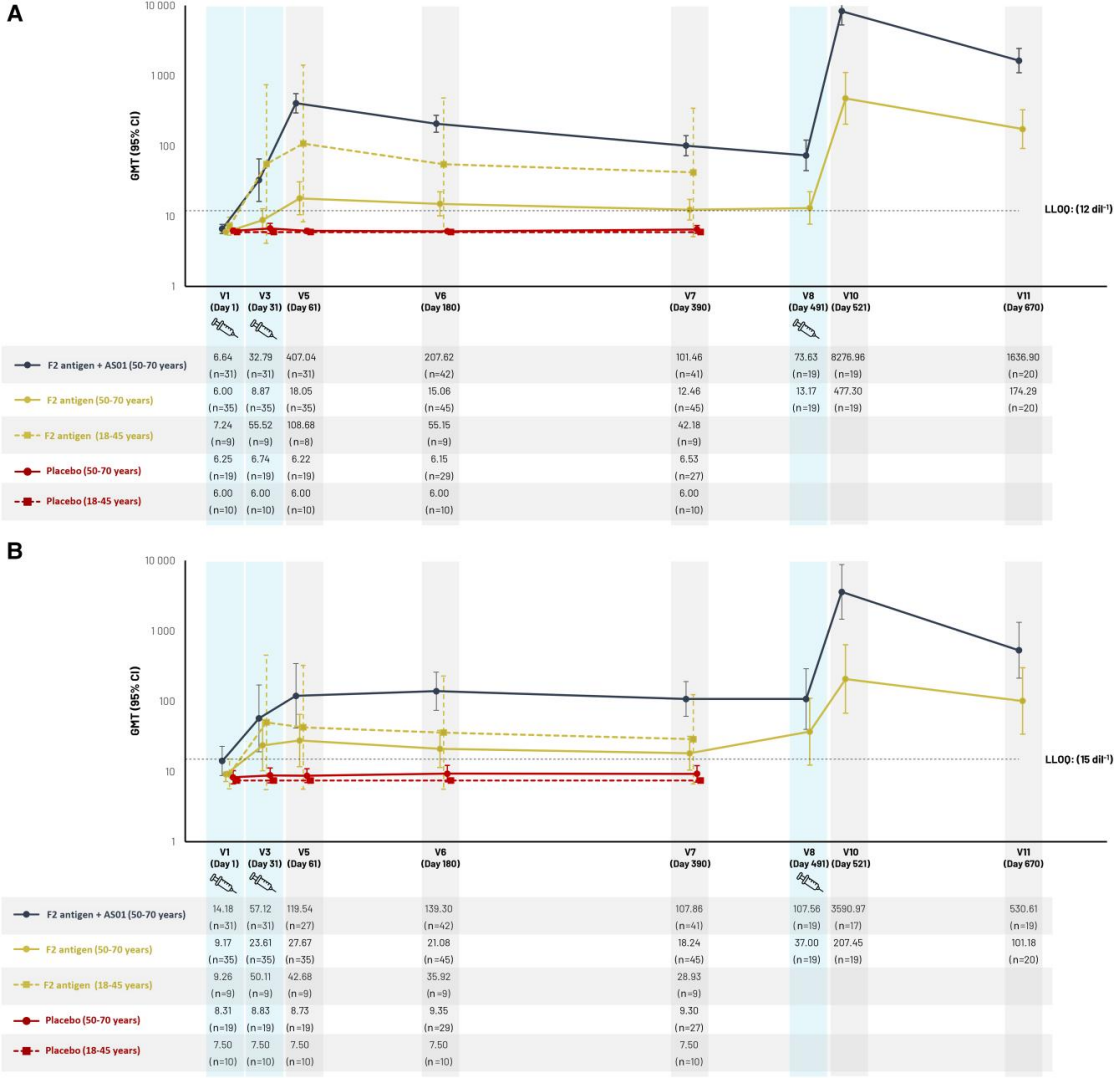
*A*, Geometric mean titer of anti-TcdA neutralization activity for subjects aged 50–70 years who received F2 antigen with or without AS01, and subjects aged 18–45 years who received F2 antigen (per-protocol set). *B*, Geometric mean titer of anti-TcdB neutralization activity for subjects aged 50–70 years who received F2 antigen with or without AS01, and subjects aged 18–45 years who received F2 antigen (per-protocol set). Abbreviations: AS01, liposome-based vaccine adjuvant system containing 2 immunostimulants: 3-*O*-desacyl-4′-monophosphoryl lipid A and the saponin QS-21; CI, confidence interval; dil, dilution; GMT, geometric mean titer; LLOQ, lower limit of quantitation; n, number of subjects with available results; TcdA, *C. difficile* toxin A; TcdB, *C. difficile* toxin B; V, visit.

Figure 3 *A* and 3*B* summarizes TcdA- and TcdB-specific neutralization activity results, respectively, in the per-protocol set. Baseline titers were <LLOQ in 77.8%–100% of subjects for TcdA neutralization activity, and in 74.2%–100% of subjects for TcdB neutralization activity, depending on the group (Supplementary Tables 7–10). In the group (aged 50–70 years) receiving F2 antigen plus AS01, TcdA neutralization GMT peaked around 400 dil^−1^ 1 month after dose 2, then declined before dose 3 but remained above baseline, increased to >8000 dil^−1^ 1 month after dose 3 and remained >1000 dil^−1^ at end of study. The decline after dose 3 appeared quicker than after dose 2 (Figure 3*A*). In the same age group receiving F2 antigen alone the pattern was similar but with lower GMT throughout. In the younger age group (18–45 years) receiving F2 antigen alone, GMT reached higher levels than in the older group (50–70 years) receiving F2 antigen alone, but not as high as obtained with the addition of AS01 in the older group (Figure 3*A*). In the group receiving F2 antigen plus AS01, TcdB neutralization GMT reached >100 dil^−1^ 1 month after dose 2, >3000 dil^−1^ 1 month after dose 3, and >500 dil^−1^ at end of study. In the same age group (50–70 years) receiving F2 antigen alone, GMT reached 27 dil^−1^ 1 month after dose 2 and >200 dil^−1^ 1 month after dose 3. After dose 3, GMT appeared to decline more rapidly than after dose 2 (Figure 3*B*). The younger age group (18–45 years) showed a response to F2 antigen alone that was higher than in the older age group receiving F2 antigen alone, but lower than in the older age group receiving F2 antigen plus AS01 (Figure 3*B*).

**Figure 4.**
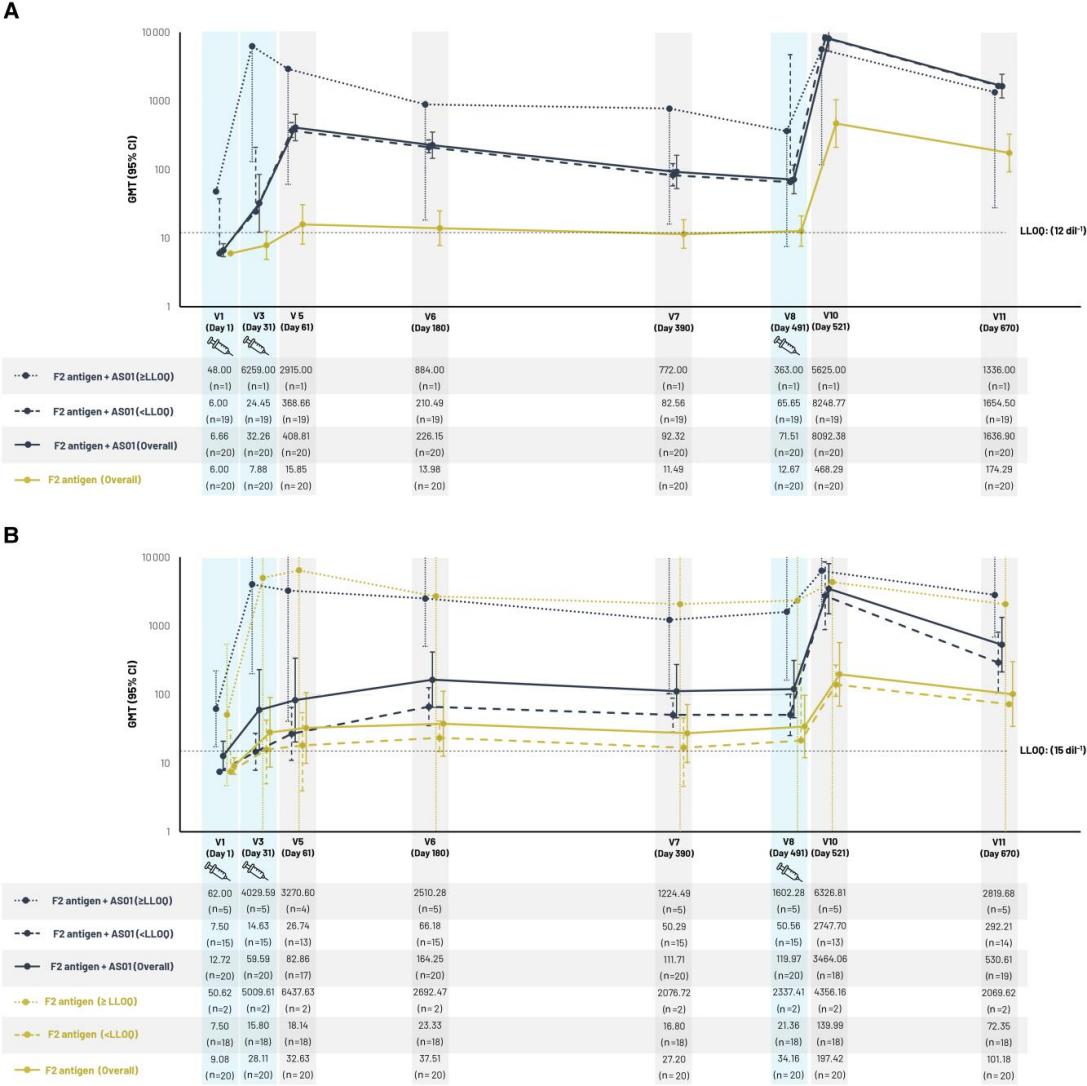
*A*, Geometric mean titer of anti-TcdA neutralization activity for subjects aged 50–70 years who received 3 doses of F2 antigen with or without AS01, (subcohort exposed set), by baseline neutralization titer. *B*, Geometric mean titer of anti-TcdB neutralization activity for subjects aged 50–70 years who received 3 doses of F2 antigen with or without AS01, (subcohort exposed set), by baseline neutralization titer. Abbreviations: AS01, liposome-based vaccine adjuvant system containing 2 immunostimulants: 3-*O*-desacyl-4′-monophosphoryl lipid A and the saponin QS-21; CI, confidence interval; dil, dilution; GMT, geometric mean titer; LLOQ, lower limit of quantitation; n, number of subjects with available results; TcdA, *C. difficile* toxin A; TcdB, *C. difficile* toxin B; V, visit.

Neutralization activity results for TcdA and TcdB split by serological baseline titers <LLOQ or ≥LLOQ are shown in Figure 4*A* and 4*B* for the subcohort receiving 3 doses and Supplementary Tables 7–10 for this subcohort and the per-protocol set. About 20% had TcdB-specific neutralization titers ≥LLOQ at baseline and reached high GMT after a single dose of F2 antigen plus AS01, with modest further increases after subsequent doses. Subjects with baseline titers <LLOQ showed moderately increased GMT after 2 doses of F2 antigen plus AS01, with a further increase in the subcohort receiving a third dose. In subjects with baseline titers ≥LLOQ receiving F2 antigen alone, GMT reached the same range as in those receiving F2 antigen plus AS01, although the number of subjects with baseline titers ≥LLOQ was small and the result should be interpreted with caution. Conversely, in subjects with baseline titers <LLOQ, administration of F2 antigen alone led to GMT values below those induced by F2 antigen plus AS01, indicating the added value of AS01 to reach high titers in subjects with baseline titers <LLOQ. Few subjects had TcdA-specific neutralization titers ≥LLOQ at baseline.

## DISCUSSION

This study was the first use in humans of the F2 antigen *C. difficile* vaccine candidate. No safety concerns were raised by the study results. The local and general AEs were mostly mild/moderate with short duration, and were more frequently reported in the adjuvanted group, as expected with the use of an adjuvant. The observed unsolicited AEs did not show any clinically relevant differences between the groups, and none of the SAEs or pIMDs were considered related to study vaccine. The results indicate that the F2 antigen vaccine candidate, with or without AS01, was well tolerated with an acceptable safety profile. TcdA- and TcdB-specific neutralization titers peaked after dose 2, then declined before dose 3 while remaining above baseline, then peaked at a higher level 1 month after dose 3 before declining to end of study levels that were still above baseline. The rate of decline after the third dose appeared faster than after the second dose, although this should be interpreted with caution given the small cohort investigated at dose 3. The pattern was broadly similar with and without AS01, although GMTs were higher with the adjuvant. There was an indication of higher responses to F2 antigen alone in the younger age group investigated (18–45 years) compared with the older age group (50–70 years), although the sample size in the younger age group was small (≤10 subjects) and a formal comparison was not part of the study design. However, this may suggest a possible age effect that could be investigated further in future research.

Other vaccine candidates against *C. difficile* have been investigated by Sanofi and Pfizer in various clinical trials, including phase 3 studies [18–25]. Development of the Sanofi vaccine has been stopped [20, 26]. A third vaccine candidate, VLA84 (Valneva), contains a recombinant fusion protein composed of portions of TcdA and TcdB, somewhat different from the sequences used in the F2 antigen (Supplementary Figure 1*B*) [27], adjuvanted with aluminum salts. The VLA84 vaccine candidate has been investigated in phase 1 and phase 2 trials [28, 29] with no development reported since completion of the phase 2 trial NCT02316470.

*C. difficile* TcdA and TcdB are internalized by gut epithelial cells and promote inflammation, tissue damage, and diarrhea [30], and TcdB may also act on pericytes and sensory neurons to induce neurogenic inflammation [31]. In human colon, TcdB is more toxic than TcdA [32], and some clinically significant strains of *C. difficile* produce TcdB and not TcdA, indicating that TcdB alone can cause disease in humans [2]. An antibody to the C-terminal portion of TcdB, bezlotoxumab, is approved as adjunctive therapy to prevent recurrent infection [2], while the effect of antibodies to the C-terminal portion of TcdA remains unclear. A pair of clinical trials found that the anti-TcdB antibody bezlotoxumab significantly reduced recurrent CDI compared with placebo, the anti-TcdA antibody actoxumab alone was not efficacious and adding actoxumab to bezlotoxumab did not improve efficacy [4]. These results suggest that TcdB has a more important role in *C. difficile* disease in humans, although TcdA may still be a contributory factor [4]. The results of the present study demonstrate that the F2 antigen-based vaccine candidate with or without AS01 is immunogenic against both TcdA and TcdB.

As similarly observed with another vaccine candidate [22], subjects with baseline neutralization titers ≥LLOQ showed stronger immune responses in the present study than subjects with baseline neutralization titers <LLOQ. Preclinical work has indicated that natural CDI is associated with a poor immune response resulting in a lack of protection against reinfection [33], and in humans the memory B-cell response to natural CDI produces antibodies with a low affinity for, and limited ability to neutralize, TcdB [34]. In the present study, administration of a third dose of F2 antigen plus AS01 elicited a substantial neutralization response in subjects with baseline titers <LLOQ, bringing these subjects up to neutralization titers comparable to those in subjects with baseline titers ≥LLOQ. Inducing a robust functional response in subjects with low baseline titers is likely to be crucial for vaccine effect as these represent most subjects in the target population. This is shown by the current study, where >80% of the healthy subjects aged 50–70 years had baseline titers <LLOQ, and also in other studies in older [22] or at-risk subjects [24]. This may indicate that a schedule including a booster dose would be needed to maximize vaccine effect.

This study has several limitations. First, the sample size was small, as is usual and appropriate for a first-in-human phase 1 study. Second, there is no generally accepted immunological correlate of protection for neutralization activity for the F2 antigen used in the *C. difficile* vaccine candidate against the *C. difficile* toxins, so the level of immunogenicity that translates into clinical efficacy is unknown. This also means that there is no recognized definition for seropositivity at baseline, which makes it difficult to assess potential effects of prior exposure to CDI. Third, the level of assay characterization and lack of standardized assays prevents comparisons between these data and results obtained from other vaccine candidates.

## CONCLUSION

The GSK *C. difficile* vaccine candidate based on F2 antigen with the AS01 adjuvant was well tolerated with an acceptable safety profile in this study. It elicited a functional humoral response to both TcdA and TcdB, and F2 antigen administered with AS01 produced a substantially higher response to both toxins than F2 antigen alone. A third dose increased the response in subjects with baseline neutralization titers <LLOQ.

## Supplementary Data

Supplementary materials are available at *The Journal of Infectious Diseases* online (http://jid.oxfordjournals.org/). Supplementary materials consist of data provided by the author that are published to benefit the reader. The posted materials are not copyedited. The contents of all supplementary data are the sole responsibility of the authors. Questions or messages regarding errors should be addressed to the author.

## Supplementary Material

jiae466_Supplementary_Data

## Notes

***Acknowledgments.*** The authors thank the study participants and the study team at the clinical trial site. IQVIA was the contract research organization for this study with funding from GSK. The authors thank Ranjan Ashutosh (IQVIA), Patricia Bourguignon (GSK at the time of study conduct), Carolina Buitrago (GSK), Nikola Bulow (GSK), Marie Dozot (GSK), Domin Ekaney (GSK), Luca Grassano (GSK), Tiffany Loy (GSK at the time of study conduct), Ana Strezova (GSK), and Elisabeth Van Eerdenbrugh (GSK) for their contributions to the study. Business and Decision Life Sciences Medical Communication Service Center provided editorial assistance and manuscript coordination, on behalf of GSK. Carole Nadin (Fleetwith, Ltd, on behalf of GSK) provided writing support. AS01 is a trademark owned by or licensed to GSK. SAS is a trademark of SAS Institute, Inc. CellTiter-Glo is a trademark of Promega.

***Author contributions.*** I. L.-R., M. C., B. d. W., M. E. I., G. S. F., A. L., S. M., C. S., and J. P. Y. contributed to the study design and methods. I. L.-R., A. A., F. D. B., and B. J. were responsible for data acquisition. I. L.-R., M. C., B. d. W., M. E. I., G. S. F., A. L., S. M., C. S., and J. P. Y. performed data analysis, and/or data interpretation. All authors read and edited the manuscript; approved the final version and the decision to submit the manuscript; had full access to all the data; and had final responsibility for the decision to submit for publication.

***Data availability.*** Anonymized individual participant data and study documents can be requested for further research from www.clinicalstudydatarequest.com.

***Financial support.*** This work was supported by GSK (GSK study identifier 208109), which was involved in all stages of study conduct, including analysis of the data. GSK also supported all costs associated with the development and publication of this article.
