# Supplementary material for: Safety and Immunogenicity of an Adjuvanted Clostridioides difficile Vaccine Candidate in Healthy Adults: A Randomized Placebo-Controlled Phase 1 Study
Source: J Infect Dis. 2024 Oct 24;231(3):e511–20. doi: 10.1093/infdis/jiae466 (PMC11911797; doi:10.1093/infdis/jiae466)
Supplement: jiae466_Supplementary_Data [file supp_material.pdf]

SUPPLEMENTARY MATERIAL

Supplementary Methods: Study population

Women of childbearing potential were eligible if they were not pregnant at the time of vaccination and were using adequate contraception for 30 days before and two months after the treatment period. Subjects with a history of *C. difficile* infection (CDI), uncontrolled chronic disease, bleeding disorders, known positivity for human immunodeficiency virus, hepatitis B virus or hepatitis C virus, immune-compromising conditions, or receipt of immunosuppressive therapy were excluded. Also excluded were subjects with acute disease and/or fever (≥38°C) at the time of vaccination, planned administration of other than protocol-specified vaccines or receiving immune-modifying/immunosuppressant drugs or corticosteroids outside the protocol-defined periods or who had received immunoglobulins within three months prior to vaccination.

Supplementary Methods: Toxin neutralisation assays

Toxin neutralisation assays (TNAs) were used to evaluate the vaccine induced responses. Serum neutralisation activity against both *C. difficile* toxin A (TcdA) and *C. difficile* toxin B (TcdB) was measured using optimised TNAs on two human colonic cell lines, chosen for their relevant tissue origin and respective sensitivity to the toxins: HT-29 cell line for TcdA TNA, and HCT-116 cell line for TcdB TNA.

HT-29 is a cell line with epithelial morphology that was isolated in 1964 from a primary tumour obtained from a 44-year-old, White, female patient with colorectal adenocarcinoma. HCT-116 cell line was isolated from the colon of an adult male with colon cancer.

All immunogenicity analyses were conducted at GSK's clinical laboratories at Rixensart, Belgium.

A brief description of the TNAs is as follows: HT-29 and HCT-116 cell lines were

cultivated under standard conditions in Dulbecco's Modified Eagle Medium (DMEM) culture medium (supplemented with heat-inactivated 10% Fetal Bovine Serum [FBS], 1% L-Glutamine and Penicillin/Streptomycin) and dispensed into white 96-well tissue culture plates and incubated overnight at 37 °C in a humidified incubator (5% CO_2). Each test serum sample was analysed separately for the ability to neutralise TcdA (HT-29) and TcdB (HCT-116), and appropriate serial dilutions of test serum samples were mixed with a fixed concentration of TcdA or TcdB and incubated at 37 °C for 90 min in a humidified incubator (37.5 °C/5% CO_2) to allow for neutralisation of the toxins to occur. For quality control, all assay runs included negative and positive controls to monitor assay performance, which consisted of human serum of known neutralisation titre. After 90 min, the serum–toxin mixtures were added to the cell monolayers, incubated at 37 °C for 6 days, and subsequently treated with a luciferase-based CellTiter-Glo reagent (Promega) to determine the adenosine triphosphate (ATP) levels measured by relative luminescence units (RLUs) in metabolically active cells. The neutralising antibody titre of the test serum sample was determined by interpolation of the reciprocal dilution which exhibited 50% reduction in cytotoxicity, as compared to live cell control (100% RLU) and toxicity control (0% RLU). The lower limits of quantitation (LLOQs) for TNAs measuring serum neutralisation activity against TcdA and TcdB were 12 dil-1 and 15 dil-1, respectively. The variability of the TNA (intermediate precision CV%) was determined to be 34% for antitoxin A and 31% for antitoxin B.

TcdA and TcdB used in the assay were purified at GSK, from culture supernatant from *C. difficile* ribotype 087. The concentration of toxin to be used in the assay was determined beforehand: Cells were dispensed to 96-well tissue culture plates at a density of 2500 HT-29 cells or 250 HCT-116 cells per well. Purified toxin was serially diluted in supplemented DMEM assay medium and added to the cells 24h after

plating. The assay plate was incubated at 37 °C for 6 days. After 6 days, cell viability was determined using the CellTiter-Glo reagent. Dose–response curves allowed definition of the amount of toxin causing 50 % reduction in luminescence, and subsequently the concentration of toxin to be used in the assay: 8xTC50.

Supplementary Figure 1. (A) F2 antigen sequence (B) VLA84 antigen sequence. VLA84 antigen sequence adapted from [1]

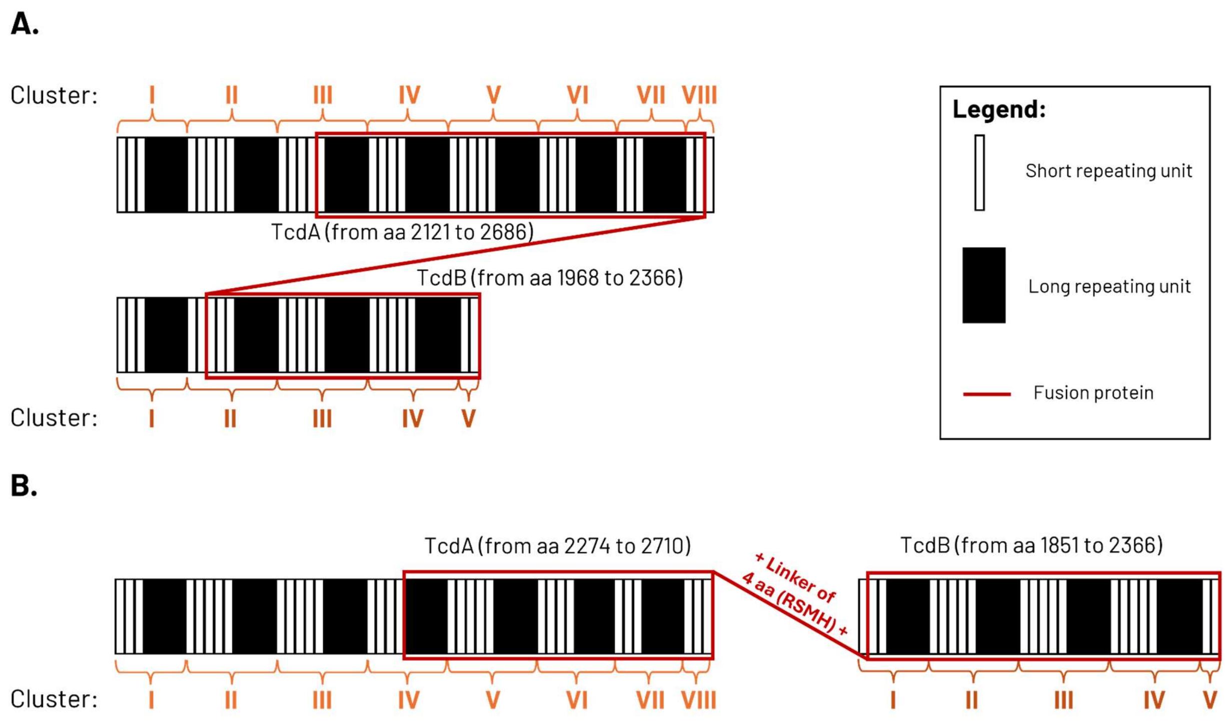

aa: amino acids; H, histidine; M, methionine; R, arginine; S, serine; TcdA, *C. difficile* toxin A; TcdB, *C. difficile* toxin B

1. Valneva Austria. WO2012028741 - NOUVEAU POLYPEPTIDE ISOLÉ DES PROTÉINES TOXINE A ET TOXINE B DE C. DIFFICILE ET UTILISATIONS ASSOCIÉES. Available at: https://patentscope.wipo.int/search/fr/detail.jsf?docId=WO2012028741.

Supplementary Figure 2. Any (A) or related (B) solicited local and general AEs reported in the 7 days after dosing (overall) (solicited safety set)

A.

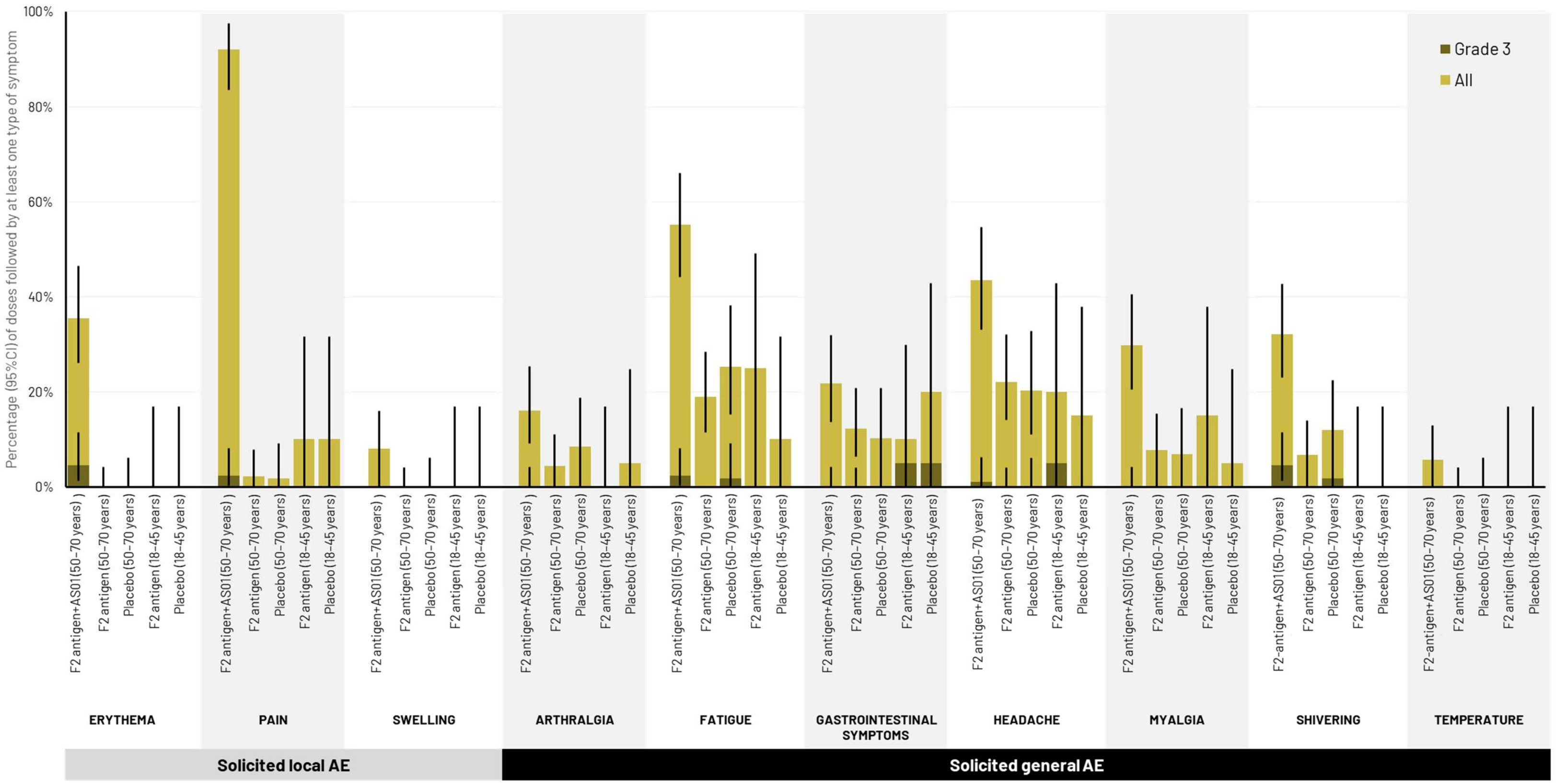

B.

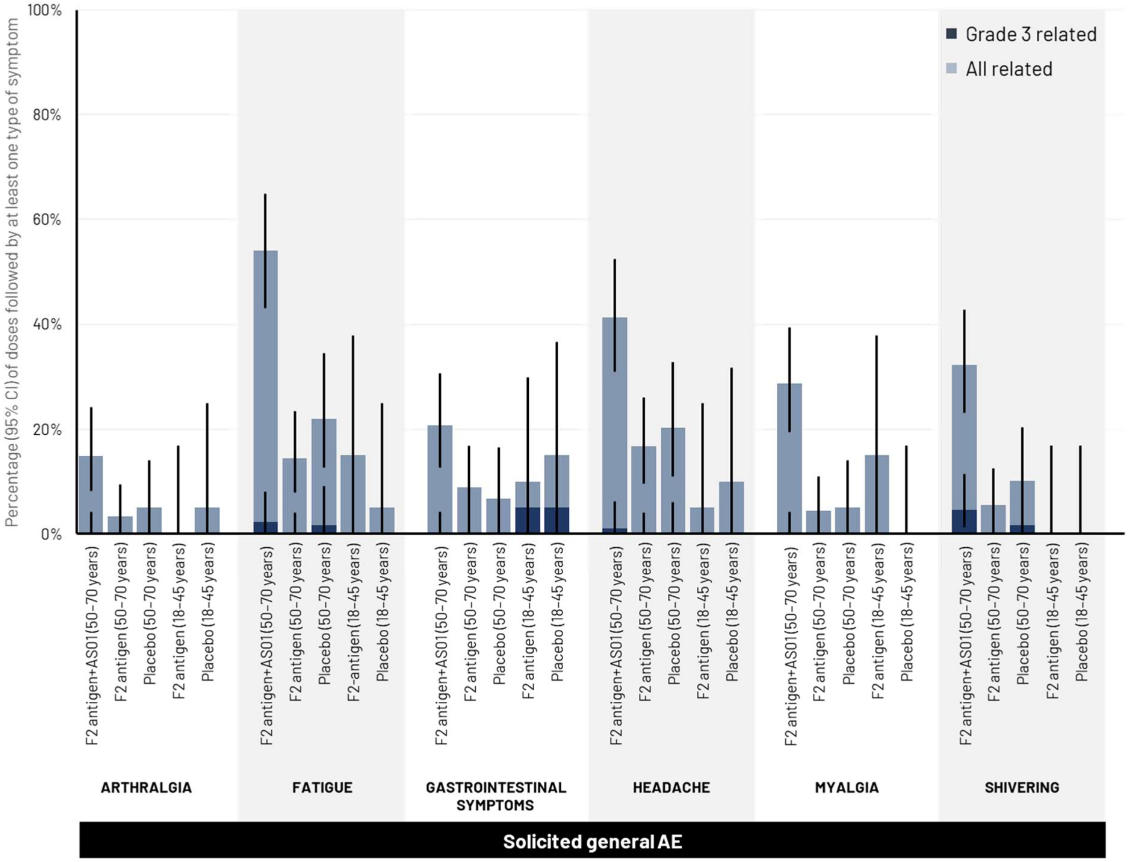

AE, adverse event; AS01, liposome-based vaccine adjuvant system containing two immunostimulants: 3-*O*-desacyl-4'-monophosphoryl lipid A (MPL) and the saponin QS-21; CI, confidence interval

Supplementary Figure 3. Any (A.) or related (B.) solicited local and general AEs reported in the 7 days after dosing (overall) (subcohort exposed set)

A.

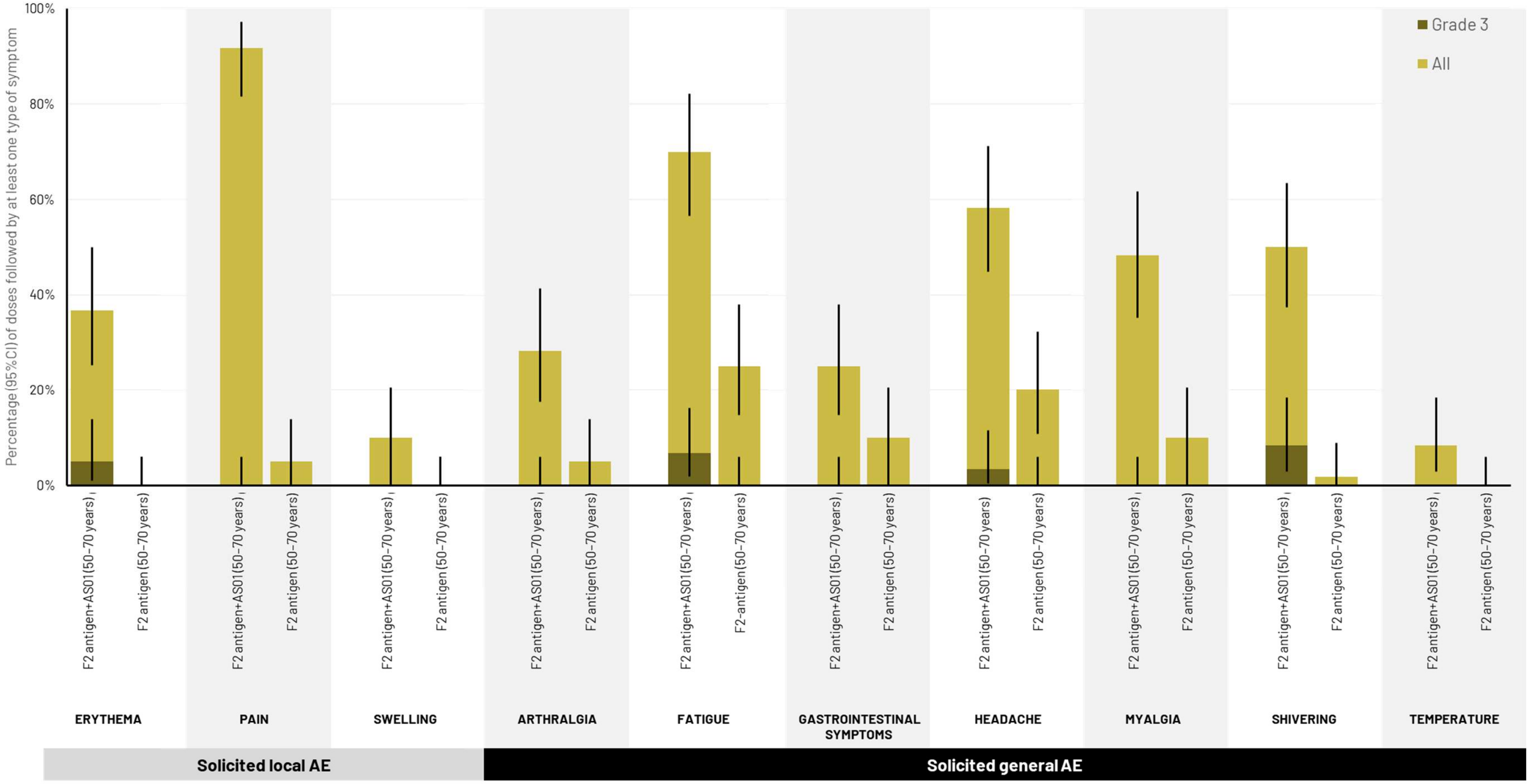

B.

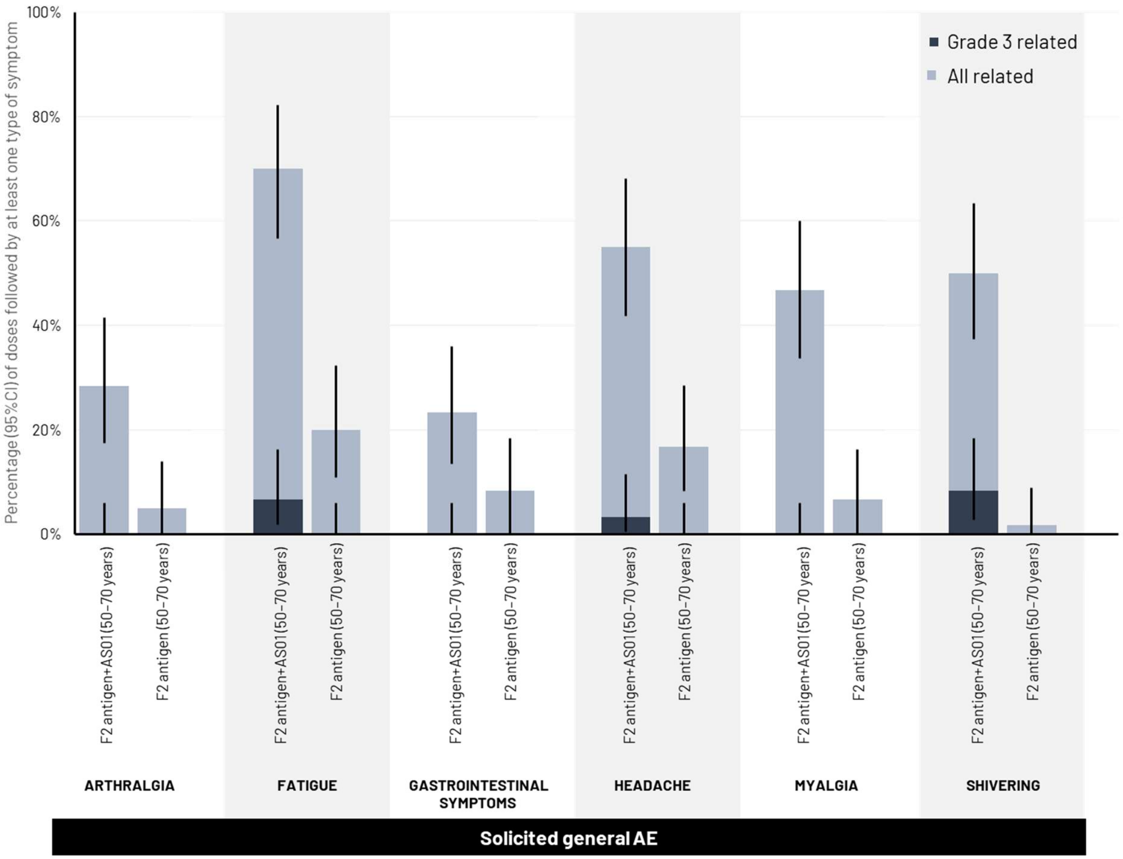

AE, adverse event; AS01, liposome-based vaccine adjuvant system containing two immunostimulants: 3-*O*-desacyl-4'-monophosphoryl lipid A (MPL) and the saponin QS-21; CI, confidence interval

Supplementary Table 1. Potential immune-mediated disorders (pIMD)

Neuroinflammatory disorders	Musculoskeletal disorders	Skin disorders
• Cranial nerve neuropathy, including paralysis and paresis (e.g., Bell's palsy).<br><br>• Optic neuritis.<br><br>• Multiple sclerosis.<br><br>• Transverse myelitis.<br><br>• Guillain-Barré syndrome, including Miller Fisher syndrome and other variants.<br><br>• Acute disseminated encephalomyelitis, including site specific variants, e.g., non-infectious encephalitis, encephalomyelitis, myelitis, myeloradiculoneuritis.<br><br>• Myasthenia gravis, including Lambert-Eaton myasthenic syndrome.<br><br>• Demyelinating peripheral neuropathies including:<br>- Chronic inflammatory demyelinating polyneuropathy.<br>- Multifocal motor neuropathy.<br>- Polyneuropathies associated with monoclonal gammopathy.<br><br>• Narcolepsy.	• Systemic lupus erythematosus and associated conditions.<br><br>• Systemic scleroderma (systemic sclerosis), including:<br>- Diffuse scleroderma.<br>- CREST syndrome.<br><br>• Idiopathic inflammatory myopathies, including:<br>- Dermatomyositis.<br>- Polymyositis.<br><br>• Antisynthetase syndrome.<br><br>• Rheumatoid arthritis and associated conditions including:<br>- Juvenile idiopathic arthritis.<br>- Still's disease.<br><br>• Polymyalgia rheumatica.<br><br>• Spondyloarthropathies, including:<br>- Ankylosing spondylitis.<br>- Reactive arthritis (Reiter's syndrome).<br>- Undifferentiated spondyloarthritis.<br>- Psoriatic arthritis.<br>- Enteropathic arthritis.<br><br>• Relapsing polychondritis.<br><br>• Mixed connective tissue disorder.<br><br>• Gout.	• Psoriasis.<br><br>• Vitiligo.<br><br>• Erythema nodosum.<br><br>• Autoimmune bullous skin diseases (including pemphigus, pemphigoid, and dermatitis herpetiformis).<br><br>• Lichen planus.<br><br>• Sweet's syndrome.<br><br>• Localized scleroderma (morphea).
**Vasculitis**	**Blood disorders**	**Others**
• Large vessels vasculitis.<br><br>including:<br>- Giant cell arteritis (temporal arteritis).<br>- Takayasu's arteritis.<br><br>• Medium sized and/or small vessels vasculitis including:	• Autoimmune hemolytic anemia.<br><br>• Autoimmune thrombocytopenia.<br><br>• Antiphospholipid syndrome.	• Autoimmune glomerulonephritis including:<br>- IgA nephropathy.<br>- Glomerulonephritis rapidly progressive.<br>- Membranous glomerulonephritis.<br>- Membranoproliferative glomerulonephritis.

- Polyarteritis nodosa.<br>- Kawasaki's disease.<br>- Microscopic polyangiitis.<br>- Wegener's granulomatosis (granulomatosis with polyangiitis).<br>- Churg–Strauss syndrome (allergic granulomatous angiitis or eosinophilic granulomatosis with polyangiitis).<br>- Buerger's disease (thromboangiitis obliterans).<br>- Necrotizing vasculitis (cutaneous or systemic).<br>- Antineutrophil cytoplasmic antibody positive vasculitis (type unspecified).<br>- Henoch-Schonlein purpura (IgA vasculitis).<br>- Behcet's syndrome.<br>- Leukocytoclastic vasculitis.	• Pernicious anemia.<br><br>• Autoimmune aplastic anemia.<br><br>• Autoimmune neutropenia.<br><br>• Autoimmune pancytopenia	- Mesangioproliferative glomerulonephritis.<br>- Tubulointerstitial nephritis and uveitis syndrome.<br><br>• Ocular autoimmune diseases including:<br>- Autoimmune uveitis.<br>- Autoimmune retinitis.<br><br>• Autoimmune myocarditis.<br><br>• Sarcoidosis.<br><br>• Stevens-Johnson syndrome.<br><br>• Sjögren's syndrome.<br><br>• Alopecia areata.<br><br>• Idiopathic pulmonary fibrosis.<br><br>• Goodpasture syndrome.<br><br>• Raynaud's phenomenon.
**Liver disorders**	**Gastrointestinal disorders**	**Endocrine disorders**
• Autoimmune hepatitis.<br><br>• Primary biliary cirrhosis.<br><br>• Primary sclerosing<br><br>cholangitis.<br><br>• Autoimmune cholangitis.	• Inflammatory bowel disease, including:<br>- Crohn's disease.<br>- Ulcerative colitis.<br>- Microscopic colitis.<br>- Ulcerative proctitis.<br><br>• Celiac disease.<br><br>• Autoimmune pancreatitis.	• Autoimmune thyroiditis (Hashimoto thyroiditis).<br><br>• Grave's or Basedow's disease.<br><br>• Diabetes mellitus type 1.<br><br>• Addison's disease.<br><br>• Polyglandular autoimmune syndrome.<br><br>• Autoimmune hypophysitis.

IgA, immunoglobulin A.

Supplementary Table 2. Demographic and baseline characteristics (exposed set)

		50-70 years			18-45 years		
		F2 antigen + AS01 (N=45)	F2 antigen (N=45)	Placebo (N=30)	F2 antigen (N=10)	Placebo (N=10)	All subjects (N=140)
**Sex**							
Male	n (%)	15 (33.3)	15 (33.3)	10 (33.3)	5 (50.0)	5 (50.0)	50 (35.7)
Female	n (%)	30 (66.7)	30 (66.7)	20 (66.7)	5 (50.0)	5 (50.0)	90 (64.3)
**Race**							
White	n (%)	45 (100.0)	45 (100.0)	30 (100.0)	10 (100.0)	10 (100.0)	140 (100.0)
**Age (years)**							
	Mean	60.8	60.7	58.9	32.7	31.5	56.2
	SD	6.0	6.1	6.2	6.8	7.7	11.7
**Age [50-69 years]**							
	n (%)	17 (37.8)	19 (42.2)	15 (50.0)	0 (0.0)	0 (0.0)	51 (42.5)
**Age [60-70 years]**							

	n (%)	28 (62.2)	26 (57.8)	15 (50.0)	0 (0.0)	0 (0.0)	69 (57.5)
**Ethnicity**							
Not Hispanic or Latino	n (%)	45 (100.0)	45 (100.0)	30 (100.0)	10 (100.0)	10 (100.0)	140 (100.0)
**BMI (kg/m^2)**							
	Mean	26.171	25.982	25.815	25.471	23.110	25.765
	SD	4.162	3.642	3.809	4.141	2.327	3.841

AS01, liposome-based vaccine adjuvant system containing two immunostimulants: 3-*O*-desacyl-4'-monophosphoryl lipid A (MPL) and the saponin QS-21; BMI, body mass index; n/%, number/percentage of subjects in each category; N, number of subjects with at least one administered dose; SD, standard deviation

Supplementary Table 3. Solicited local and general AEs reported in the 7 days after dosing (overall) (solicited safety set)

AE	F2 antigen + AS01 50–70 years (N=87)		F2 antigen 50–70 years (N=90)		Placebo 50–70 years (N=59)		F2 antigen 18–45 years (N=20)		Placebo 18–45 years (N=20)	
	**n (%)**	**95% CI**	**n (%)**	**95% CI**	**n (%)**	**95% CI**	**n (%)**	**95% CI**	**n (%)**	**95% CI**
**Local AEs**										
**Erythema**										
All	**27** (31.0)	21.5, 41.9	**0**	0.0, 4.0	**0**	0.0, 6.1	**0**	0.0, 16.8	**0**	0.0, 16.8
Grade 3	**4** (4.6)	1.3, 11.4	**0**	0.0, 4.0	**0**	0.0, 6.1	**0**	0.0, 16.8	**0**	0.0, 16.8
**Pain**										
All	**78** (89.7)	81.3, 95.2	**2** (2.2)	0.3, 7.8	**1.7**	0.0, 9.1	**2** (10.0)	1.2, 31.7	**2** (10.0)	1.2, 31.7
Grade 3	**2** (2.3)	0.3, 8.1	**0**	0.0, 4.0	**0**	0.0, 6.1	**0**	0.0, 16.8	**0**	0.0, 16.8
**Swelling**										
All	**7** (8.0)	3.3, 15.9	**0**	0.0, 4.0	**0**	0.0, 6.1	**0**	0.0, 16.8	**0**	0.0, 16.8
Grade 3	**0**	0.0, 4.2	**0**	0.0, 4.0	**0**	0.0, 6.1	**0**	0.0, 16.8	**0**	0.0, 16.8
**General AEs**										
**Arthralgia**										
All	**14** (16.1)	9.1, 25.5	**4** (4.4)	1.2, 11.0	**5** (8.5)	2.8, 18.7	**0**	0.0, 16.8	**1** (5.0)	0.1, 24.9

AE	F2 antigen + AS01 50–70 years (N=87)		F2 antigen 50–70 years (N=90)		Placebo 50–70 years (N=59)		F2 antigen 18–45 years (N=20)		Placebo 18–45 years (N=20)	
	**n (%)**	**95% CI**	**n (%)**	**95% CI**	**n (%)**	**95% CI**	**n (%)**	**95% CI**	**n (%)**	**95% CI**
Grade 3	**0**	0.0, 4.2	**0**	0.0, 4.0	**0**	0.0, 6.1	**0**	0.0, 16.8	**0**	0.0, 16.8
Related	**13** (14.9)	8.2, 24.2	**3** (3.3)	0.7, 9.4	**3** (5.1)	1.1, 14.1	**0**	0.0, 16.8	**1** (5.0)	0.1, 24.9
Grade 3, related	**0**	0.0, 4.2	**0**	0.0, 4.0	**0**	0.0, 6.1	**0**	0.0, 16.8	**0**	0.0, 16.8
**Fatigue**										
All	**46** (52.9)	41.9, 63.7	**17** (18.9)	11.4, 28.5	**14** (23.7)	13.6, 36.6	**5** (25.0)	8.7, 49.1	**2** (10.0)	1.2, 31.7
Grade 3	**2** (2.3)	0.3, 8.1	**0**	0.0, 4.0	**1** (1.7)	0.0, 9.1	**0**	0.0, 16.8	**0**	0.0, 16.8
Related	**45** (51.7)	40.8, 62.6	**13** (14.4)	7.9, 23.4	**12** (20.3)	11.0, 32.8	**3** (15.0)	32.0, 37.9	**1** (5.0)	0.1, 24.9
Grade 3, related	**2** (2.3)	0.3, 8.1	**0**	0.0, 4.0	**1** (1.7)	0.0, 9.1	**0**	0.0, 16.8	**0**	0.0, 16.8
**Gastrointestinal symptoms**										
All	**19** (21.8)	13.7, 32.0	**11** (12.2)	6.3, 20.8	**6** (10.2)	3.8, 20.8	**1** (5.0)	0.1, 24.9	**3** (15.0)	3.2, 37.9
Grade 3	**0**	0.0, 4.2	**0**	0.0, 4.0	**0**	0.0, 6.1	**1** (5.0)	0.1, 24.9	**1** (5.0)	0.1, 24.9
Related	**18** (20.7)	12.7, 30.7	**8** (8.9)	3.9, 16.8	**4** (6.8)	1.9, 16.5	**1** (5.0)	0.1, 24.9	**2** (10.0)	1.2, 31.7
Grade 3, related	**0**	0.0, 4.2	**0**	0.0, 4.0	**0**	0.0, 6.1	**1** (5.0)	0.1, 24.9	**1** (5.0)	0.1, 24.9
**Headache**										

AE	F2 antigen + AS01 50–70 years (N=87)		F2 antigen 50–70 years (N=90)		Placebo 50–70 years (N=59)		F2 antigen 18–45 years (N=20)		Placebo 18–45 years (N=20)	
	**n (%)**	**95% CI**	**n (%)**	**95% CI**	**n (%)**	**95% CI**	**n (%)**	**95% CI**	**n (%)**	**95% CI**
All	**37** (42.5)	32.0, 53.6	**20** (22.2)	14.1, 32.2	**12** (20.3)	11.0, 32.8	**3** (15.0)	3.2, 37.9	**3** (15.0)	3.2, 37.9
Grade 3	**1** (1.1)	0.0, 6.2	**0**	0.0, 4.0	**0**	0.0, 6.1	**1** (5.0)	0.1, 24.9	**0**	0.0, 16.8
Related	**35** (40.2)	29.9, 51.3	**15** (16.7)	9.6, 26.0	**12** (20.3)	11.0, 32.8	**1** (5.0)	0.1, 24.9	**2** (10.0)	1.2, 31.7
Grade 3, related	**1** (1.1)	0.0, 6.2	**0**	0.0, 4.0	**0**	0.0, 6.1	**0**	0.0, 16.8	**0**	0.0, 16.8
**Myalgia**										
All	**26** (29.9)	20.5, 40.6	**7** (7.8)	3.2, 15.4	**4** (6.8)	1.9, 16.5	**3** (15.0)	3.2, 37.9	**1** (5.0)	0.1, 24.9
Grade 3	**0**	0.0, 4.2	**0**	0.0, 4.0	**0**	0.0, 6.1	**0**	0.0, 16.8	**0**	0.0, 16.8
Related	**25** (28.7)	19.5, 39.4	**4** (4.4)	1.2, 11.0	**3** (5.1)	1.1, 14.1	**3** (15.0)	3.2, 37.9	**0**	0.0, 16.8
Grade 3, related	**0**	0.0, 4.2	**0**	0.0, 4.0	**0**	0.0, 6.1	**0**	0.0, 16.8	**0**	0.0, 16.8
**Shivering**										
All	**24** (27.6)	18.5, 38.2	**6** (6.7)	2.5, 13.9	**6** (10.2)	3.8, 20.8	**0**	0.0, 16.8	**0**	0.0, 16.8
Grade 3	**4** (4.6)	1.3, 11.4	**0**	0.0, 4.0	**1** (1.7)	0.0, 9.1	**0**	0.0, 16.8	**0**	0.0, 16.8
Related	**24** (27.6)	18.5, 38.2	**5** (5.6)	1.8, 12.5	**5** (8.5)	2.8, 18.7	**0**	0.0, 16.8	**0**	0.0, 16.8
Grade 3, related	**4** (4.6)	1.3, 11.4	**0**	0.0, 4.0	**1** (1.7)	0.0, 9.1	**0**	0.0, 16.8	**0**	0.0, 16.8

AE	F2 antigen + AS01 50–70 years (N=87)		F2 antigen 50–70 years (N=90)		Placebo 50–70 years (N=59)		F2 antigen 18–45 years (N=20)		Placebo 18–45 years (N=20)	
	**n (%)**	**95% CI**	**n (%)**	**95% CI**	**n (%)**	**95% CI**	**n (%)**	**95% CI**	**n (%)**	**95% CI**
**Temperature**										
All	**5** (5.7)	1.9, 12.9	**0**	0.0, 4.0	**0**	0.0, 6.1	**0**	0.0, 16.8	**0**	0.0, 16.8
>40.0°C	**0**	0.0, 4.2	**0**	0.0, 4.0	**0**	0.0, 6.1	**0**	0.0, 16.8	**0**	0.0, 16.8
>40.0°C, related	**0**	0.0, 4.2	**0**	0.0, 4.0	**0**	0.0, 6.1	**0**	0.0, 16.8	**0**	0.0, 16.8

AE, adverse event; AS01, liposome-based vaccine adjuvant system containing two immunostimulants: 3-*O*-desacyl-4'-monophosphoryl lipid A (MPL) and the saponin QS-21; CI, confidence interval; N, number of documented doses; n (%), number/percentage of doses followed by at least one type of symptom.

Supplementary Table 4. Median duration of solicited AEs (overall) (exposed set)

	F2 antigen + AS01 50–70 years		F2 antigen 50–70 years		Placebo 50–70 years		F2 antigen 18–45 years		Placebo 18–45 years	
**AE**	**N**	**Median (days)**	**N**	**Median (days)**	**N**	**Median (days)**	**N**	**Median (days)**	**N**	**Median (days)**
**Local AEs**										
Redness	32	**3.0**	6	**1.0**	2	**1.0**	2	**2.5**	0	**ND**
Pain	78	**3.0**	2	**1.0**	1	**1.0**	2	**1.0**	2	**1.0**
Swelling	25	**2.0**	2	**2.5**	2	**1.5**	0	**ND**	0	**ND**
**General AEs**										
Arthralgia	14	**1.0**	4	**2.0**	5	**2.0**	0	**ND**	1	**1.0**
Fatigue	46	**2.0**	17	**3.0**	14	**3.0**	5	**2.0**	2	**2.5**
Fever	5	**1.0**	0	**ND**	0	**ND**	0	**ND**	0	**ND**
Gastrointestinal symptoms	19	**1.0**	11	**1.0**	6	**1.5**	1	**3.0**	3	**1.0**
Headache	37	**1.0**	20	**1.0**	12	**1.5**	3	**2.0**	3	**1.0**
Myalgia	26	**1.5**	7	**2.0**	4	**2.0**	3	**1.0**	1	**1.0**
Shivering	24	**1.0**	6	**1.0**	6	**1.5**	0	**ND**	0	**ND**

AE, adverse event; AS01, liposome-based vaccine adjuvant system containing two immunostimulants: 3-*O*-desacyl-4’-monophosphoryl lipid A (MPL) and the saponin QS-21; N, number of doses with the symptom; ND, not determined.

Supplementary Table 5. Solicited local and general AEs reported in the 7 days after dosing (overall) (subcohort exposed set)

	F2 antigen + AS01<br>50–70 years (N=60)		F2 antigen<br>50–70 years (N=60)	
**AE**	**n (%)**	**95% CI**	**n (%)**	**95% CI**
**Local AEs**				
**Erythema**				
All	**19** (31.7)	20.3, 45.0	**0**	0.0, 6.0
Grade 3	**3** (5.0)	1.0, 13.9	**0**	0.0, 6.0
**Pain**				
All	**55** (91.7)	81.6, 97.2	**3** (5.0)	1.0, 13.9
Grade 3	**0**	0.0, 6.0	**0**	0.0, 6.0
**Swelling**				
All	**6** (10.0)	3.8, 20.5	**0**	0.0, 6.0
Grade 3	**0**	0.0, 6.0	**0**	0.0, 6.0
**General AEs**				
**Arthralgia**				
All	**17** (28.3)	17.5, 41.4	**3** (5.0)	1.0, 13.9

AE	F2 antigen + AS01<br><br>50–70 years (N=60)		F2 antigen<br><br>50–70 years (N=60)	
	**n (%)**	**95% CI**	**n (%)**	**95% CI**
Grade 3	**0**	0.0, 6.0	**0**	0.0, 6.0
Related	**17** (28.3)	17.5, 41.4	**3** (5.0)	1.0, 13.9
Grade 3, related	**0**	0.0, 6.0	**0**	0.0, 6.0
**Fatigue**				
All	**38** (63.3)	49.9, 75.4	**15** (25.0)	14.7, 37.9
Grade 3	**4** (6.7)	1.8, 16.2	**0**	0.0, 6.0
Related	**38** (63.3)	49.9, 75.4	**12** (20.0)	10.8, 32.3
Grade 3, related	**4** (6.7)	1.8, 16.2	**0**	0.0, 6.0
**Gastrointestinal symptoms**				
All	**15** (25.0)	14.7, 37.9	**6** (10.0)	3.8, 20.5
Grade 3	**0**	0.0, 6.0	**0**	0.0, 6.0
Related	**14** (23.3)	13.4, 36.0	**5** (8.3)	2.8, 18.4
Grade 3, related	**0**	0.0, 6.0	**0**	0.0, 6.0
**Headache**				

AE	F2 antigen + AS01<br>50–70 years (N=60)		F2 antigen<br>50–70 years (N=60)	
	**n (%)**	**95% CI**	**n (%)**	**95% CI**
All	**33** (55.0)	41.6, 67.9	**12** (20.0)	10.8, 32.3
Grade 3	**2** (3.3)	0.4. 11.5	**0**	0.0, 6.0
Related	**31** (51.7)	38.4, 64.8	**10** (16.7)	8.3, 28.5
Grade 3, related	**2** (3.3)	0.4. 11.5	**0**	0.0, 6.0
**Myalgia**				
All	**29** (48.3)	35.2, 61.6	**6** (10.0)	3.8, 20.5
Grade 3	**0**	0.0, 6.0	**0**	0.0, 6.0
Related	**28** (46.7)	33.7, 60.0	**4** (6.7)	1.8, 16.2
Grade 3, related	**0**	0.0, 6.0	**0**	0.0, 6.0
**Shivering**				
All	**25** (41.7)	29.1, 55.1	**1** (1.7)	0.0, 8.9
Grade 3	**5** (8.3)	2.8, 18.4	**0**	0.0, 6.0
Related	**25** (41.7)	29.1, 55.1	**1** (1.7)	0.0, 8.9
Grade 3, related	**5** (8.3)	2.8, 18.4	**0**	0.0, 6.0

AE	F2 antigen + AS01<br>50–70 years (N=60)		F2 antigen<br>50–70 years (N=60)	
	**n (%)**	**95% CI**	**n (%)**	**95% CI**
**Temperature**				
All	**5** (8.3)	2.8, 18.4	**0**	0.0, 6.0
>40.0°C	**0**	0.0, 6.0	**0**	0.0, 6.0
>40.0°C, related	**0**	0.0, 6.0	**0**	0.0, 6.0

AE, adverse event; AS01, liposome-based vaccine adjuvant system containing two immunostimulants: 3-*O*-desacyl-4'-monophosphoryl lipid A (MPL) and the saponin QS-21; CI, confidence interval; N, number of documented doses; n (%), number/percentage of doses followed by at least one type of symptom.

Supplementary Table 6. Median duration of solicited AEs (overall) (subcohort exposed set)

AE	F2 antigen + AS01 50–70 years		F2 antigen 50–70 years	
	**N**	**Median (days)**	**N**	**Median (days)**
**Local AEs**				
Redness	23	**3.0**	2	**3.5**
Pain	55	**3.0**	3	**1.0**
Swelling	20	**2.0**	1	**4.0**
**General AEs**				
Arthralgia	17	**2.0**	3	**4.0**
Fatigue	38	**2.0**	15	**3.0**
Fever	5	**1.0**	0	**ND**
Gastrointestinal symptoms	15	**2.0**	6	**1.5**
Headache	33	**2.0**	12	**1.0**
Myalgia	29	**2.0**	6	**4.0**
Shivering	25	**1.0**	1	**1.0**

AE, adverse event; AS01, liposome-based vaccine adjuvant system containing two immunostimulants: 3-*O*-desacyl-4'-monophosphoryl lipid A (MPL) and the saponin QS-21; N, number of doses with the symptom; ND, not determined.

Supplementary Table 7. Number of subjects with TcdA-specific (HT-29) neutralisation titres ≥ 12 and geometric mean titres, by baseline activity < or ≥ LLOQ and age cohort (Per-protocol set)

					GMT		
**Group**	**Time**	**Status at baseline**	**N**	**n**	**Value**	**95% CI LL**	**95% CI UL**
**F2 antigen + AS01 50-70 years**	**Visit 1 (Day 1)**	≥ LLOQ	2	2	**28.57**	0.04	20,882.13
		< LLOQ	29	0	**6.00**	/	/
		Overall	31	2	**6.64**	5.70	7.72
	**Visit 3 (Day 31)**	≥ LLOQ	2	2	**2993.81**	0.26	35,138,200.93
		< LLOQ	29	17	**24.02**	13.55	42.59
		Overall	31	19	**32.79**	16.34	65.80
	**Visit 5 (Day 61)**	≥ LLOQ	2	2	**2002.76**	17.00	235,975.94
		< LLOQ	29	29	**364.69**	272.44	488.17
		Overall	31	31	**407.04**	296.97	557.92
	**Visit 6 (Day 180)**	≥ LLOQ	2	2	**791.12**	193.06	3241.92
		< LLOQ	40	40	**194.19**	147.71	255.29
		Overall	42	42	**207.62**	157.44	273.80
	**Visit 7 (Day 390)**	≥ LLOQ	2	2	**524.24**	3.84	71,653.75
		< LLOQ	39	38	**93.27**	67.47	128.93
		Overall	41	40	**101.46**	72.90	141.22
	**Visit 8 (Day 491)**	≥ LLOQ	1	1	**363.00**	/	/
		< LLOQ	18	17	**67.39**	41.13	110.40
		Overall	19	18	**73.63**	44.62	121.51
	**Visit 10 (Day 521)**	≥ LLOQ	1	1	**5625.00**	/	
		< LLOQ	18	18	**8456.49**	5271.20	13,566.59
		Overall	19	19	**8276.96**	5290.84	12,948.43

	**Visit 11 (Day 670)**	≥ LLOQ	1	1	**1336.00**	/	/
		< LLOQ	19	19	**1654.50**	1087.34	2517.47
		Overall	20	20	**1636.90**	1100.16	2435.51
**F2 antigen 50-70 years**	**Visit 1 (Day 1)**	< LLOQ	35	0	**6.00**	/	/
		Overall	35	0	**6.00**	/	/
	**Visit 3 (Day 31)**	< LLOQ	35	5	**8.87**	6.13	12.83
		Overall	35	5	**8.87**	6.13	12.83
	**Visit 5 (Day 61)**	< LLOQ	35	13	**18.05**	10.54	30.93
		Overall	35	13	**18.05**	10.54	30.93
	**Visit 6 (Day 180)**	< LLOQ	45	17	**15.06**	10.17	22.32
		Overall	45	17	**15.06**	10.17	22.32
	**Visit 7 (Day 390)**	< LLOQ	45	15	**12.46**	8.86	17.52
		Overall	45	15	**12.46**	8.86	17.52
	**Visit 8 (Day 491)**	< LLOQ	19	8	**13.17**	7.76	22.36
		Overall	19	8	**13.17**	7.76	22.36
	**Visit 10 (Day 521)**	< LLOQ	19	19	**477.30**	204.95	1111.53
		Overall	19	19	**477.30**	204.95	1111.53
	**Visit 11 (Day 670)**	< LLOQ	20	20	**174.29**	92.64	327.90
		Overall	20	20	**174.29**	92.64	327.90
**Placebo 50-70 years**	**Visit 1 (Day 1)**	≥ LLOQ	1	1	**13.00**	/	/
		< LLOQ	18	0	**6.00**	/	/
		Overall	19	1	**6.25**	5.74	6.81
	**Visit 3 (Day 31)**	≥ LLOQ	1	1	**18.00**	/	/
		< LLOQ	18	1	**6.38**	5.61	7.25
		Overall	19	2	**6.74**	5.70	7.96
	**Visit 5 (Day 61)**	≥ LLOQ	1	1	**12.00**	/	/
		< LLOQ	18	0	**6.00**	/	/

		Overall	19	1	**6.22**	5.76	6.72
	**Visit 6 (Day 180)**	≥ LLOQ	1	1	**12.00**	/	/
		< LLOQ	28	0	**6.00**	/	/
		Overall	29	1	**6.15**	5.85	6.45
	**Visit 7 (Day 390)**	≥ LLOQ	1	1	**12.00**	/	/
		< LLOQ	26	1	**6.38**	5.62	7.25
		Overall	27	2	**6.53**	5.73	7.45
**F2 antigen 18-45 years**	**Visit 1 (Day 1)**	≥ LLOQ	2	2	**13.96**	5.63	34.66
		< LLOQ	7	0	**6.00**	/	/
		Overall	9	2	**7.24**	5.43	9.65
	**Visit 3 (Day 31)**	≥ LLOQ	2	2	**4963.60**	0.01	1,978,119,291.12
		< LLOQ	7	1	**15.38**	1.54	153.91
		Overall	9	3	**55.52**	4.14	743.92
	**Visit 5 (Day 61)**	≥ LLOQ	2	2	**3602.89**	0.02	592,701,632.64
		< LLOQ	6	3	**33.83**	2.43	470.95
		Overall	8	5	**108.68**	8.35	1414.69
	**Visit 6 (Day 180)**	≥ LLOQ	2	2	**2149.12**	0.03	166,706,062.07
		< LLOQ	7	3	**19.37**	2.68	139.98
		Overall	9	5	**55.15**	6.32	481.00
	**Visit 7 (Day 390)**	≥ LLOQ	2	2	**1584.15**	0.31	8,092,044.55
		< LLOQ	7	2	**14.97**	2.26	99.08
		Overall	9	4	**42.18**	5.16	344.79
**Placebo 18-45 years]**	**Visit 1 (Day 1)**	< LLOQ	10	0	**6.00**	/	/
		Overall	10	0	**6.00**	/	/
	**Visit 3 (Day 31)**	< LLOQ	10	0	**6.00**	/	/
		Overall	10	0	**6.00**	/	/
	**Visit 5 (Day 61)**	< LLOQ	10	0	**6.00**	/	/

		Overall	10	0	**6.00**	/	/
	**Visit 6 (Day 180)**	< LLOQ	10	0	**6.00**	/	/
		Overall	10	0	**6.00**	/	/
	**Visit 7 (Day 390)**	< LLOQ	10	0	**6.00**	/	/
		Overall	10	0	**6.00**	/	/

AS01, liposome-based vaccine adjuvant system containing two immunostimulants: 3-*O*-desacyl-4’-monophosphoryl lipid A (MPL) and the saponin QS-21; CI, confidence interval; GMT, geometric mean antibody titres; LL, lower limit; UL, upper limit; LLOQ, lower limit of quantitation; N, number of subjects with available results; n, number of subjects with concentration equal to or above specified cut-off.

Supplementary Table 8. Number of subjects with TcdA-specific (HT-29) neutralisation titres ≥ 12 and geometric mean titres, by baseline activity < or ≥ LLOQ and age cohort (subcohort exposed set)

					GMT		
**Group**	**Time**	**Status at baseline**	**N**	**n**	**Value**	**95% CI LL**	**95% CI UL**
**F2 antigen + AS01 50-70 years**	**Visit 1 (Day 1)**	≥ LLOQ	1	1	**48.00**	/	/
		< LLOQ	19	0	**6.00**	/	/
		Overall	20	1	**6.66**	5.36	8.28
	**Visit 3 (Day 31)**	≥ LLOQ	1	1	**6259.00**	/	/
		< LLOQ	19	10	**24.45**	10.71	55.81
		Overall	20	11	**32.26**	12.20	85.29
	**Visit 5 (Day 61)**	≥ LLOQ	1	1	**2915.00**	/	/
		< LLOQ	19	19	**368.66**	244.96	544.82
		Overall	20	20	**408.81**	262.55	636.56
	**Visit 6 (Day 180)**	≥ LLOQ	1	1	**884.00**	/	/
		< LLOQ	19	19	**210.49**	136.43	324.77
		Overall	20	20	**226.15**	146.16	349.92
	**Visit 7 (Day 390)**	≥ LLOQ	1	1	**772.00**	/	/
		< LLOQ	19	18	**82.56**	48.08	141.77
		Overall	20	19	**92.32**	52.63	161.95
	**Visit 8 (Day 491)**	≥ LLOQ	1	1	**363.00**	/	/
		< LLOQ	19	18	**65.65**	41.10	104.85
		Overall	20	19	**71.51**	44.36	115.26
	**Visit 10 (Day 521)**	≥ LLOQ	1	1	**5625.00**	/	/
		< LLOQ	19	19	**8248.77**	5268.72	12914.38
		Overall	20	20	**8092.38**	5287.56	12,385.01

	**Visit 11 (Day 670)**	≥ LLOQ	1	1	**1336.00**	/	/
		< LLOQ	19	19	**1654.50**	1087.34	2517.47
		Overall	20	20	**1636.90**	1100.16	2435.51
**F2 antigen 50-70 years**	**Visit 1 (Day 1)**	< LLOQ	20	0	**6**	/	/
		Overall	20	0	**6**	/	/
	**Visit 3 (Day 31)**	< LLOQ	20	2	**7.88**	4.91	12.64
		Overall	20	2	**7.88**	4.91	12.64
	**Visit 5 (Day 61)**	< LLOQ	20	7	**15.85**	8.14	30.85
		Overall	20	7	**15.85**	8.14	30.85
	**Visit 6 (Day 180)**	< LLOQ	20	7	**13.98**	7.82	24.98
		Overall	20	7	**13.98**	7.82	24.98
	**Visit 7 (Day 390)**	< LLOQ	20	7	**11.49**	7.1	18.61
		Overall	20	7	**11.49**	7.1	18.61
	**Visit 8 (Day 491)**	< LLOQ	20	8	**12.67**	7.63	21.02
		Overall	20	8	**12.67**	7.63	21.02
	**Visit 10 (Day 521)**	< LLOQ	20	20	**468.29**	210.42	1042.14
		Overall	20	20	**468.29**	210.42	1042.14
	**Visit 11 (Day 670)**	< LLOQ	20	20	**174.29**	92.64	327.90
		Overall	20	20	**174.29**	92.64	327.90

AS01, liposome-based vaccine adjuvant system containing two immunostimulants: 3-*O*-desacyl-4'-monophosphoryl lipid A (MPL) and the saponin QS-21; CI, confidence interval; GMT, geometric mean antibody titres; LL, lower limit; UL, upper limit; LLOQ, lower limit of quantitation; N, number of subjects with available results; n, number of subjects with concentration equal to or above specified cut-off.

Supplementary Table 9. Number of subjects with TcdB-specific (HT-116) neutralisation titres ≥ 15 and geometric mean titres, by baseline activity < or ≥ LLOQ and age cohort (per-protocol set)

					GMT		
**Group**	**Time**	**Status at baseline**	**N**	**n**	**Value**	**95% CI LL**	**95% CI UL**
**F2 antigen + AS01 50-70 years**	**Visit 1 (Day 1)**	≥ LLOQ	8	8	**88.58**	26.26	298.75
		< LLOQ	23	0	**7.50**	/	/
		Overall	31	8	**14.18**	8.79	22.88
	**Visit 3 (Day 31)**	≥ LLOQ	8	8	**5090.36**	937.26	27,646.28
		< LLOQ	23	6	**11.98**	7.94	18.07
		Overall	31	14	**57.12**	19.16	170.24
	**Visit 5 (Day 61)**	≥ LLOQ	7	7	**4386.04**	552.59	34,813.07
		< LLOQ	20	15	**33.88**	18.23	62.95
		Overall	27	22	**119.54**	41.49	344.42
	**Visit 6 (Day 180)**	≥ LLOQ	11	11	**2041.69**	919.95	4531.20
		< LLOQ	31	27	**53.73**	34.80	82.96
		Overall	42	38	**139.30**	74.75	259.60
	**Visit 7 (Day 390)**	≥ LLOQ	11	11	**1097.56**	363.99	3309.52
		< LLOQ	30	28	**46.07**	33.03	64.26
		Overall	41	39	**107.86**	60.93	190.93
	**Visit 8 (Day 491)**	≥ LLOQ	4	4	**1824.05**	64.57	51,525.69
		< LLOQ	15	13	**50.56**	25.15	101.66
		Overall	19	17	**107.56**	39.93	289.77
	**Visit 10 (Day 521)**	≥ LLOQ	4	4	**8570.37**	2535.84	28,965.25
		< LLOQ	13	13	**2747.70**	878.89	8590.20
		Overall	17	17	**3590.97**	1468.26	8782.55

	**Visit 11 (Day 670)**	≥ LLOQ	5	5	**2819.68**	685.68	11,595.22
		< LLOQ	14	14	**292.21**	105.18	811.83
		Overall	19	19	**530.61**	212.57	1324.49
**F2 antigen 50-70 years**	**Visit 1 (Day 1)**	≥ LLOQ	3	3	**78.11**	11.41	534.53
		< LLOQ	32	0	**7.50**	/	/
		Overall	35	3	**9.17**	7.23	11.63
	**Visit 3 (Day 31)**	≥ LLOQ	3	3	**4851.11**	95.58	24,6221.68
		< LLOQ	32	5	**14.33**	7.39	27.78
		Overall	35	8	**23.61**	10.21	54.61
	**Visit 5 (Day 61)**	≥ LLOQ	3	3	**5993.48**	152.70	235,239.62
		< LLOQ	32	7	**16.71**	8.45	33.05
		Overall	35	10	**27.67**	11.78	64.99
	**Visit 6 (Day 180)**	≥ LLOQ	3	3	**2361.67**	169.23	32,958.25
		< LLOQ	42	9	**15.05**	9.11	24.87
		Overall	45	12	**21.08**	11.48	38.73
	**Visit 7 (Day 390)**	≥ LLOQ	3	3	**1773.77**	46.75	67,305.43
		< LLOQ	42	8	**13.15**	8.60	20.12
		Overall	45	11	**18.24**	10.51	31.64
	**Visit 8 (Day 491)**	≥ LLOQ	2	2	**2337.41**	0.00	11,722,574,197,373.40
		< LLOQ	17	6	**22.72**	9.31	55.45
		Overall	19	8	**37.00**	12.39	110.53
	**Visit 10 (Day 521)**	≥ LLOQ	2	2	**4356.16**	0.00	24,481,941,063.71
		< LLOQ	17	15	**145.00**	48.10	437.07
		Overall	19	17	**207.45**	67.61	636.54
	**Visit 11 (Day 670)**	≥ LLOQ	2	2	**2069.62**	0.00	2,094,182,197,164,515.00
		< LLOQ	18	16	**72.35**	25.90	202.10
		Overall	20	18	**101.18**	34.17	299.64

**Placebo 50-70 years**	**Visit 1 (Day 1)**	≥ LLOQ	1	1	**53.00**	/	/
		< LLOQ	18	0	**7.50**	/	/
		Overall	19	1	**8.31**	6.70	10.32
	**Visit 3 (Day 31)**	≥ LLOQ	1	1	**50.00**	/	/
		< LLOQ	18	1	**8.02**	6.96	9.23
		Overall	19	2	**8.83**	6.93	11.25
	**Visit 5 (Day 61)**	≥ LLOQ	1	1	**46.00**	/	/
		< LLOQ	18	1	**7.96**	7.02	9.03
		Overall	19	2	**8.73**	6.96	10.96
	**Visit 6 (Day 180)**	≥ LLOQ	2	2	**92.69**	0.02	396,785.12
		< LLOQ	27	1	**7.89**	7.11	8.74
		Overall	29	3	**9.35**	7.15	12.22
	**Visit 7 (Day 390)**	≥ LLOQ	2	2	**83.89**	0.15	46,798.01
		< LLOQ	25	1	**7.80**	7.19	8.46
		Overall	27	3	**9.30**	7.12	12.15
**F2 antigen 18-45 years**	**Visit 1 (Day 1)**	≥ LLOQ	1	1	**50.00**	/	/
		< LLOQ	8	0	**7.50**	/	/
		Overall	9	1	**9.26**	5.70	15.06
	**Visit 3 (Day 31)**	≥ LLOQ	1	1	**3793.00**	/	/
		< LLOQ	8	2	**29.17**	3.53	240.91
		Overall	9	3	**50.11**	5.54	453.47
	**Visit 5 (Day 61)**	≥ LLOQ	1	1	**3151.00**	/	/
		< LLOQ	8	2	**24.93**	3.86	161.09
		Overall	9	3	**42.68**	5.62	324.34
	**Visit 6 (Day 180)**	≥ LLOQ	1	1	**2717.00**	/	/
		< LLOQ	8	2	**20.92**	4.27	102.43
		Overall	9	3	**35.92**	5.65	228.40

	Visit 7 (Day 390)	≥ LLOQ	1	1	**1228.00**	/	/
		< LLOQ	8	3	**18.11**	5.76	56.96
		Overall	9	4	**28.93**	6.70	124.87
**Placebo 18-45 years**	**Visit 1 (Day 1)**	< LLOQ	10	0	**7.50**	/	/
		Overall	10	0	**7.50**	/	/
	**Visit 3 (Day 31)**	< LLOQ	10	0	**7.50**	/	/
		Overall	10	0	**7.50**	/	/
	**Visit 5 (Day 61)**	< LLOQ	10	0	**7.50**	/	/
		Overall	10	0	**7.50**	/	/
	**Visit 6 (Day 180)**	< LLOQ	10	0	**7.50**	/	/
		Overall	10	0	**7.50**	/	/
	**Visit 7 (Day 390)**	< LLOQ	10	0	**7.50**	/	/
		Overall	10	0	**7.50**	/	/

AS01, liposome-based vaccine adjuvant system containing two immunostimulants: 3-*O*-desacyl-4'-monophosphoryl lipid A (MPL) and the saponin QS-21; CI, confidence interval; GMT, geometric mean antibody titres; LL, lower limit; UL, upper limit; LLOQ, lower limit of quantitation; N, number of subjects with available results; n, number of subjects with concentration equal to or above specified cut-off.

Supplementary Table 10. Number of subjects with TcdB-specific (HT-116) neutralisation titres ≥ 15 and geometric mean titres, by baseline activity < or ≥ LLOQ and age cohort (subcohort exposed set)

					GMT		
**Group**	**Time**	**Status at baseline**	**N**	**n**	**Value**	**95% CI LL**	**95% CI UL**
**F2 antigen + AS01 50-70 years**	**Visit 1 (Day 1)**	≥ LLOQ	5	5	**62.00**	17.38	221.22
		< LLOQ	15	0	**7.50**	/	/
		Overall	20	5	**12.72**	7.78	20.78
	**Visit 3 (Day 31)**	≥ LLOQ	5	5	**4029.59**	200.95	80,803.27
		< LLOQ	15	5	**14.63**	7.85	27.24
		Overall	20	10	**59.59**	15.36	231.10
	**Visit 5 (Day 61)**	≥ LLOQ	4	4	**3270.60**	40.59	263,536.42
		< LLOQ	13	8	**26.74**	10.96	65.25
		Overall	17	12	**82.86**	20.31	338.01
	**Visit 6 (Day 180)**	≥ LLOQ	5	5	**2510.28**	502.23	12,546.90
		< LLOQ	15	15	**66.18**	35.02	125.07
		Overall	20	20	**164.25**	64.90	415.65
	**Visit 7 (Day 390)**	≥ LLOQ	5	5	**1224.49**	102.81	14,584.23
		< LLOQ	15	14	**50.29**	28.50	88.74
		Overall	20	19	**111.71**	45.83	272.30
	**Visit 8 (Day 491)**	≥ LLOQ	5	5	**1602.28**	162.86	15,763.89
		< LLOQ	15	13	**50.56**	25.15	101.66
		Overall	20	18	**119.97**	45.75	314.59
	**Visit 10 (Day 521)**	≥ LLOQ	5	5	**6326.81**	1948.24	20,546.00
		< LLOQ	13	13	**2747.70**	878.89	8590.20
		Overall	18	18	**3464.06**	1491.57	8044.99

	**Visit 11 (Day 670)**	≥ LLOQ	5	5	**2819.68**	685.68	11,595.22
		< LLOQ	14	14	**292.21**	105.18	811.83
		Overall	19	19	**530.61**	212.57	1324.49
**F2 antigen 50-70 years**	**Visit 1 (Day 1)**	≥ LLOQ	2	2	**50.62**	4.73	542.01
		< LLOQ	18	0	**7.50**	/	/
		Overall	20	2	**9.08**	6.88	11.97
	**Visit 3 (Day 31)**	≥ LLOQ	2	2	**5009.61**	0.00	2,617,199,270,021.84
		< LLOQ	18	4	**15.80**	6.49	38.48
		Overall	20	6	**28.11**	8.70	90.74
	**Visit 5 (Day 61)**	≥ LLOQ	2	2	**6437.63**	0.00	855,826,184,463.90
		< LLOQ	18	5	**18.14**	7.35	44.74
		Overall	20	7	**32.63**	9.95	107.05
	**Visit 6 (Day 180)**	≥ LLOQ	2	2	**2692.47**	0.01	1,411,956,821.41
		< LLOQ	18	6	**23.33**	9.12	59.69
		Overall	20	8	**37.51**	12.59	111.77
	**Visit 7 (Day 390)**	≥ LLOQ	2	2	**2076.72**	0.00	178,976,362,087.37
		< LLOQ	18	5	**16.80**	8.19	34.46
		Overall	20	7	**27.20**	10.33	71.64
	**Visit 8 (Day 491)**	≥ LLOQ	2	2	**2337.41**	0.00	11,722,574,197,373.40
		< LLOQ	18	6	**21.36**	9.16	49.84
		Overall	20	8	**34.16**	11.98	97.40
	**Visit 10 (Day 521)**	≥ LLOQ	2	2	**4356.16**	0.00	24,481,941,063.71
		< LLOQ	18	16	**139.99**	49.58	395.25
		Overall	20	18	**197.42**	68.08	572.51
	**Visit 11 (Day 670)**	≥ LLOQ	2	2	**2069.62**	0.00	2,094,182,197,164,515.00
		< LLOQ	18	16	**72.35**	25.90	202.10
		Overall	20	18	**101.18**	34.17	299.64

AS01, liposome-based vaccine adjuvant system containing two immunostimulants: 3-*O*-desacyl-4'-monophosphoryl lipid A (MPL) and the saponin QS-21; CI, confidence interval; GMT, geometric mean antibody titres; LL, lower limit; UL, upper limit; LLOQ, lower limit of quantitation; N, number of subjects with available results; n, number of subjects with concentration equal to or above specified cut-off.
